# It’s about time! Exploring time allocation patterns of adults with lower literacy skills on a digital assessment

**DOI:** 10.3389/fpsyg.2024.1338014

**Published:** 2024-06-07

**Authors:** Gal Kaldes, Elizabeth L. Tighe, Qiwei He

**Affiliations:** ^1^Adult Literacy Research Center, College of Education and Human Development, Georgia State University, Atlanta, GA, United States; ^2^Department of Psychology, Georgia State University, Atlanta, GA, United States; ^3^Data Science and Analytics Program, Georgetown University, Washington, DC, United States

**Keywords:** adult literacy, PIAAC, digital literacy, behavioral process data, assessment

## Abstract

**Introduction:**

Despite the necessity for adults with lower literacy skills to undergo and succeed in high-stakes computer-administered assessments (e.g., GED, HiSET), there remains a gap in understanding their engagement with digital literacy assessments.

**Methods:**

This study analyzed process data, specifically time allocation data, from the Program for the International Assessment of Adult Competencies (PIAAC), to investigate adult respondents’ patterns of engagement across all proficiency levels on nine digital literacy items. We used cluster analysis to identify distinct groups with similar time allocation patterns among adults scoring lower on the digital literacy assessment. Finally, we employed logistic regression to examine whether the groups varied by demographic factors, in particular individual (e.g., race/ethnicity, age) and contextual factors (e.g., skills-use at home).

**Results:**

Adults with lower literacy skills spent significantly less time on many of the items than adults with higher literacy skills. Among adults with lower literacy skills, two groups of time allocation patterns emerged: one group (Cluster 1) exhibited significantly longer engagement times, whereas the other group (Cluster 2) demonstrated comparatively shorter durations. Finally, we found that adults who had a higher probability of Cluster 1 membership (spending more time) exhibited relatively higher literacy scores, higher self-reported engagement in writing skills at home, were older, unemployed, and self-identified as Black.

**Discussion:**

These findings emphasize differences in digital literacy engagement among adults with varying proficiency levels. Additionally, this study provides insights for the development of targeted interventions aimed at improving digital literacy assessment outcomes for adults with lower literacy skills.

## Introduction

One in five adults in the U.S. (19%) perform at the lowest levels of literacy, and an additional 33% are nearing but not quite at proficient literacy levels (NCES, 2019). Many of these adults attend literacy programs to achieve adequate reading, writing, and math skills necessary for attaining a high school equivalency degree, joining the workforce (or elevating their career), or continuing into postsecondary education. Adult literacy students must oftentimes engage in high-stakes assessments for various reasons. For example, adult literacy students who enroll to attain a high school equivalency degree must take and ultimately pass assessments, such as the GED and HiSET. Additionally, adult literacy programs use information from high-stakes assessments to monitor progress, make funding decisions, and determine student educational levels (Lesgold and Welch-Ross, 2012).

One important challenge related to understanding the performance of adults with lower reading skills on high-stakes assessments is that they demonstrate diversity across an array of individual differences, including demographics, educational attainment, and foundational reading skills (Lesgold and Welch-Ross, 2012; MacArthur et al., 2012; Talwar et al., 2020; Tighe et al., 2023a). An added challenge is that high-stakes assessments have become increasingly digital (e.g., GED, HiSET). Some adults with lower literacy skills demonstrate stronger basic information and communication technology (ICT) skills than others (Olney et al., 2017), suggesting that this population is heterogeneous in terms of their technology skills and experiences.

Previous literature has considered heterogeneity in ICT performance amongst adults who struggle with literacy skills; however, little attention has been paid to how adults with lower literacy skills respond to and engage with digital assessments. Understanding how adults interact with digital assessments is crucial because challenges with reading and processing information in addition to knowing and adapting to technological features may influence their performance on these assessments. Process data (log files) from large-scale datasets could provide insight into various testing behaviors in a digital testing environment, such as the time spent on items (e.g., He and von Davier, 2016; He et al., 2019; Liao et al., 2019), which may offer a more nuanced understanding of the challenges that adults with lower literacy skills face within the context of taking digital assessments. It is also anticipated that such testing behaviors may vary across different individual and contextual factors with this population, including self-reported ICT skills-use, level of education, age, race/ethnicity, native language status, self-reported learning disability status, and literacy proficiency levels (Lesgold and Welch-Ross, 2012; Wicht et al., 2021). The purpose of this study was to use extant data from a large-scale digital assessment, Program for the International Assessment of Adult Competencies (PIAAC), to understand the time allocation patterns of adults of varying literacy levels on digital literacy performance and whether this varies by individual and contextual factors.

## Digital assessment performance of adults with lower literacy skills

Recent literature has proposed a framework which posits that ICT skills on digital assessments vary amongst adults as a facet of numerous *individual* (e.g., literacy skills, education, and other socio-demographic characteristics) and *contextual* factors (i.e., the environments in which adults engage in and are exposed to ICT skills; Wicht et al., 2021).

Adults with lower literacy skills tend to struggle with basic ICT skills, which are vital to engaging with assessments in digital environments (Olney et al., 2017); yet may also demonstrate variability in skills within the realm of technology (Lesgold and Welch-Ross, 2012). For example, Olney et al. (2017) reported that adult literacy students between third to eighth grade reading levels demonstrated the highest degrees of difficulty on tasks that involved simple typing (e.g., signing into emails, composing emails) and right-clicking. However, the authors also found that the students demonstrated varying degrees of difficulty with various tasks ranging from basic computer skills to using web-features in a simulated email environment.

### Individual factors

Adults who struggle with literacy skills represent a diverse population and therefore, may exhibit differences in assessment performance across a range of literacy levels, educational, and demographic factors (Lesgold and Welch-Ross, 2012). Preliminary evidence from paper-based reading assessments with adults who exhibit lower literacy skills has reported different profiles of literacy performance that also varied by age and native language status (e.g., MacArthur et al., 2012; Talwar et al., 2020). In addition, Tighe et al. (2022) reported that adults with lower literacy levels demonstrated the lowest performance on a paper-based literacy assessment when they exhibited lower educational attainment, self-reported a learning disability, were non-native English speakers, self-reported fair/poor health status, had lower or unknown parental education levels, and identified as Hispanic ethnicity.

Literacy assessments have increasingly become digital, which places higher demands on adults with lower literacy skills because they must have the reading and writing skills to succeed on the assessment as well as the technological skills necessary to meet the demands of the varying item types (Brinkley-Etzkorn and Ishitani, 2016; Graesser et al., 2019). Previous literature posits that literacy skills, specifically the ability to decode and comprehend written language, are a precursor to adults’ ICT skills (Coiro, 2003; van Deursen and van Dijk, 2016; Iñiguez-Berrozpe and Boeren, 2020; Wicht et al., 2021). Specific to digital assessment performance, Wicht et al., 2021 found that that adults’ literacy skills were positively associated with their performance on a digital assessment that required various literacy, ICT, and problem-solving skills. Few studies to date have focused on the relation of literacy skills to adults’ ICT skills and ICT use (de Haan, 2003; van Deursen and van Dijk, 2016; Wicht et al., 2021). Previous evidence with adults who exhibit lower literacy skills suggests that their performance on literacy assessments varies (Talwar et al., 2020), thus additional work is needed to understand the relation between literacy levels and engagement with digital assessments with this population.

### Contextual factors

Practice engagement theory (PET) emphasizes that adults learn by engaging in day-to-day activities in reading, writing, and numeracy at home and at work (Reder, 2009a, 2010). This view of learning is particularly relevant to adults with lower literacy skills because they must read for various purposes in daily life (e.g., reading news stories, writing emails, filling out forms), and may encounter different types of texts, including texts in digital formats (OECD, 2013; Trawick, 2019). PET also posits that social practices play an integral role in the development of ICT skills for current cohorts of adults, especially older adults, because many of them did not formally receive ICT skills-training (Wicht et al., 2021). Instead, many of them depend on “learning-by-doing” in both home and work environments (Helsper and Eynon, 2010; Wicht et al., 2021).

Moreover, some evidence suggests that ICT skills-use in daily life varies amongst adults who have lower literacy skills (Feinberg et al., 2019), which is consistent with the heterogeneous nature of this population (Lesgold and Welch-Ross, 2012). For example, Feinberg et al. (2019) found a strong relation between performance on the PIAAC literacy assessment and self-reported frequencies of writing emails at home and at work, such that adults with relatively higher literacy scores were also more inclined to engage in ICT skills-use (e.g., writing emails) in daily life.

## Using behavioral data to understand digital assessment performance

In addition to individual differences and contextual factors, it is critical to understand the behaviors of adults with lower literacy skills as they engage with digital assessments, which may reveal additional information regarding the strategies that they use or the challenges that they may face throughout the task (Cromley and Azevedo, 2006). As such, these behaviors may provide additional information about their performance beyond their overall, global score on a digital literacy assessment. Unlike individual factors (e.g., race/ethnicity, age, native speaker status), engagement in educational tasks and settings is not a static trait of the individual and may change in-the-moment for various reasons, such as the nature or difficulty of the task (Hadwin et al., 2001), the reader’s perceived value of the task, and/or the reader’s self-efficacy while engaged in the task (Dietrich et al., 2016; Shernoff et al., 2016).

In particular, understanding the length of time adults with lower literacy skills spend immersed in a digital assessment may reveal additional insights regarding their performance. Broadly, the children’s literature suggests that reading amount, or time spent reading, is related to students’ intrinsic motivation (Wang and Guthrie, 2004; Durik et al., 2006). Moreover, sustained engagement is important to successfully integrating and coordinating multiple cognitive processes, such as decoding, vocabulary, making inferences, and comprehension monitoring (Guthrie and Wigfield, 2017). Specific to adults with lower literacy skills, evidence from eye-tracking data suggests that readers who spent more time engaging and reading key areas of the text were more likely to correctly score on reading comprehension questions and were better at summarizing and explaining key information in text (Tighe et al., 2023a). In addition, Fang et al. (2018) found different performance clusters of adults with low literacy skills based on accuracy and timing data from interacting with a digital learning environment.

Process data (log files) can provide important behavioral information about respondents’ engagement in a digital assessment (Cromley and Azevedo, 2006; He and von Davier, 2015, 2016; Fang et al., 2018; He et al., 2019; Liao et al., 2019). Process data is informative for understanding how respondents engage in a digital assessment because it provides the amount of time spent performing specific actions, including the amount of time spent until the respondent initiated an action on an item (e.g., highlighting, choosing/clicking on an answer) or the amount of time that occurred between different actions (e.g., time it took between choosing an answer [last action] and moving onto the next item). Such timing data offers nuanced information regarding the amount of time adults with lower reading skills spent reading and processing the information in the test item before choosing an answer or submitting their response. Moreover, previous research with PIAAC data suggests that these behaviors are linked to response accuracy on digital problem-solving items, and that differences exist across certain individual and contextual factors (He and von Davier, 2015, 2016; He et al., 2019; Liao et al., 2019). For example, Liao et al. (2019) found that adults who were well-educated, had higher ICT skills-use, and reported more numeracy skills at work spent more time on digital assessment items that required higher reading skills, planning, and problem-solving. Similarly, adults with lower literacy skills are heterogeneous in terms of individual (e.g., educational attainment, age, learning disability status) and contextual (e.g., ICT, reading, and writing skills-use) characteristics, which may relate to time they spend on a digital assessment. Moreover, the literature reports mixed results regarding whether more time on digital assessments is beneficial for adult test-takers. Some research suggests that test-takers with higher skills need less time to successfully respond to assessment items (e.g., van Der Linden, 2007; Klein Entink, 2009; Wang and Xu, 2015; Fox and Marianti, 2016); however, other evidence suggests that test-takers can benefit from spending more time (Klein Entink et al., 2009). Therefore, additional work with adults who have lower literacy skills is needed to understand the relation between digital literacy performance and time spent on the assessment.

## Current study

The current study used demographic and process data from the PIAAC to investigate three research questions. First, we examined whether adults with lower literacy skills (Levels 2 and below on the literacy scale) demonstrated different patterns of time allocation on a digital literacy assessment (i.e., time to performing the first action, time for the last action, and total time) from adults who demonstrate higher literacy skills. Because previous studies suggest more time is indicative of higher literacy scores (e.g., Liao et al., 2019), we anticipated that overall, adults with higher literacy skills would show patterns of increased time on digital literacy items. Second, this study investigated whether adult respondents with lower literacy skills (who scored Levels 2 and below on the literacy scale) were heterogeneous in terms of the amount of time they spent on a digital literacy assessment. Previous studies with adults who have lower literacy skills suggest that this population is diverse in terms of their reading skills and various individual, demographic characteristics (e.g., MacArthur et al., 2012; Talwar et al., 2020; Tighe et al., 2022), thus we anticipated that adults with lower literacy skills may also reveal different patterns of time spent on a digital assessment. Specifically, we expected that some of the respondents would spend substantially more time on digital literacy items than others. Finally, we examined whether different groups of individuals who demonstrated distinct patterns of time allocation varied according to multiple individual (self-reported learning disability status, employment status, educational attainment, age, native English-speaking status, race/ethnicity, literacy scores) and contextual (ICT, reading, writing, and numeracy skills-use) factors. We expected that adults with lower literacy skills who spent substantially more time on the digital literacy items would also score higher on the literacy scale and report higher skills-use. We specifically asked the following research questions:

1. Do adults with lower literacy skills on the PIAAC literacy scale (Levels 2 and below) demonstrate different patterns of time allocation on digital literacy items compared to adults who scored higher on the literacy scale?
2. Can we identify distinct clusters, or groups, of adults with lower literacy skills that emerge from the time allocation data?
3. Do individual and contextual factors predict identified cluster timing membership for adults with lower literacy skills?

## Methods

This study analyzed behavioral data from the PIAAC public log files, specifically focusing on the computer-based version of the PIAAC literacy domain assessment. We investigated the timing of respondents who took the easiest items (Testlet L11) in stage 1 of the literacy assessment across three timing variables: time to first action, time for last action, and total time. For individuals scoring at Levels 2 and below, we used cluster analysis to examine potential groupings on the nine items across all three timing variables. Finally, we used demographic characteristics to predict cluster membership, including skills-use (ICT, reading, writing, numeracy), age, native English speaker status, self-reported learning disability status, educational attainment, employment status, and race/ethnicity.

## Participants

Behavioral data (time to the first action, time for last action, total time) were available on 2,697 U.S. adults (ages 16–65) from the public PIAAC log files (process data; OECD, 2017). Depending on the specific testlet, 843–967 adults were available. Across testlets L11, L12, and L13, the sample consisted of 44.7% males, 87.1% native English speakers, and 70.4% of the sample self-identified as White. Adults with lower literacy skills (Levels 2 and below) represented 38 to 43% of the respondents across the three testlets.

## Measures

### General PIAAC survey

The PIAAC survey was developed to assess the proficiency of cognitive and workplace competencies of adults. Participants completed an extensive background questionnaire, literacy, and numeracy domains. Some participants also received a problem-solving in technology rich environments (PSTRE) domain and a paper-based reading components supplement (OECD, 2013). For this study, we only focus on the literacy domain and demographics and skills-use variables (ICT, reading, writing, and numeracy) from the background questionnaire.

### Literacy domain

Content and questions for the literacy domain were derived from previous large-scale international assessments that were administered to adults [e.g., International Adult Literacy Survey (IALS)]. This domain tapped underlying cognitive skills and applied literacy skills that were deemed necessary to meet the demands of adults living and working during the 21st century (PIAAC Literacy Expert Group, 2009).

The PIAAC literacy domain included a range of texts (e.g., electronic and narrative texts across different medias). The assessment scores are reported on a 500-point scale, which fall into 5 different literacy proficiency levels that are characterized by different reading skills: In the first proficiency level, Below Level 1 (0–175 points), respondents can locate specific information in brief, familiar texts with minimal competing information. Basic vocabulary skills are sufficient, and literacy tasks are not specific to digital text features. In Level 1 (176–225 points), respondents can read short texts in various formats to locate information, and basic vocabulary skills are essential. In Level 2 (226–275 points), respondents can match text information, make low-level inferences, and sometimes navigate texts presented in a digital medium. In Level 3 (276–325 points), respondents can understand dense or lengthy texts, make inferences, and navigate complex digital texts. In Level 4 (326–375 points), respondents can perform multi-step operations, interpret complex information, and handle competing data. In Level 5 (376–500 points), respondents are able to perform tasks that demand integrating information across multiple dense texts, construct syntheses, and evaluate evidence-based arguments.

**Figure 1:**
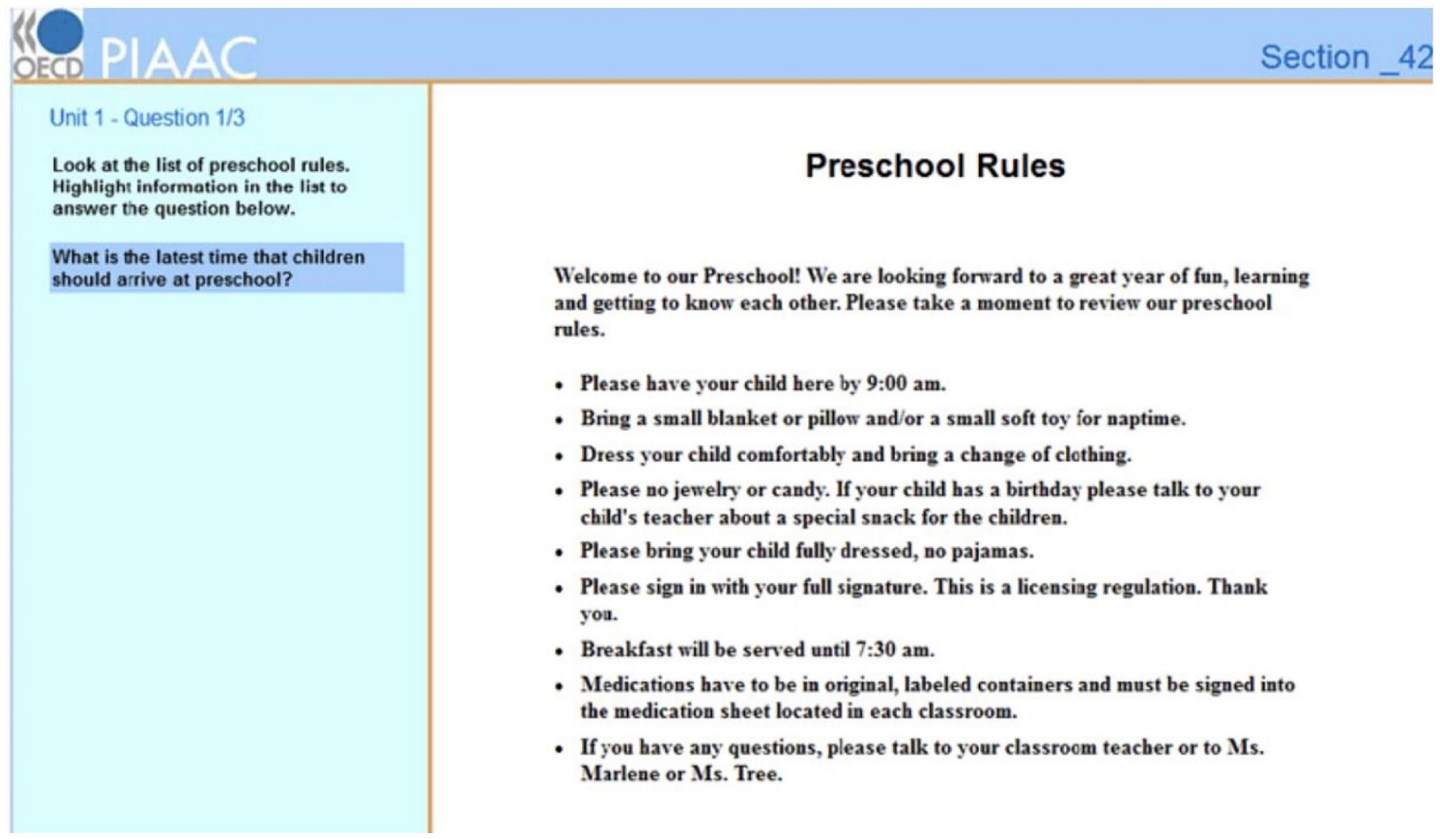
Sample Level 3 Literacy Item. This sample item was available from the technical report of the survey of adult skills (PIAAC, OECD, 2013).

Item assignment on the literacy domain assessment followed a multistage adaptive testing rule. Each literacy module consisted of 20 items distributed across two sessions: nine items in stage 1 and 11 items in stage 2 (see Figure 1 for a sample literacy item). Some participants took a paper-based literacy assessment and thus were not included in the analysis for this study.

It is noted that there was a total of 18 unique literacy items included in stage 1 of the PIAAC literacy assessment. These 18 items were assigned into three testlets (L11, L12, and L13) following an integrated block design.^1^ As a result, each testlet consisted of nine items (OECD, 2017). Details of item assignment in the three testlets are reported in Supplementary Table S1. These three testlets varied in difficulty: easy (L11), medium (L12), and difficult (L13), with an average difficulty level of −0.218, 0.485, and 0.972, respectively.^2^

The assignment of each respondent to a testlet was determined by three crucial variables: (a) education level (EdLevel3), categorized as low, medium, or high; (b) native versus nonnative speaker status, considering respondents as native speakers if their first language belonged to the assessment languages; and (c) the CBA-Core Stage 2 screening score. These variables were structured into a matrix, yielding two threshold numbers (refer to Supplementary Table S2 for a sample of the matrix design for testlet selection). Following assignment rules, respondents with a low education level, nonnative speaker status, and a low score on the CBA-Core Stage 2 screening were more likely to be assigned to the easier testlet, Testlet L11.

Despite these assignment criteria, the distribution of respondents with lower literacy levels (Levels 2 and below) remained notably similar across the testlets. Specifically, in Testlet L11, identified as the easiest testlet, 43% of respondents were categorized as Levels 2 and below. Comparatively, Testlets L12 and L13 had 44 and 38% of respondents at Levels 2 and below, respectively (see Supplementary Table S3 for a detailed breakdown of the frequency of respondents across levels in each testlet).

For research questions 2 and 3 of the present study, which primarily target respondents scoring Levels 2 and below, we focused more closely on Testlet L11. The items in Testlet L11 are acknowledged as the simplest items within the PIAAC literacy domain and were deliberately chosen to correspond with the proficiency levels of respondents who were more likely to score lower on the assessment. Consequently, conducting a thorough examination of timing in Testlet L11, with its easier items, enabled us to offer a more precise and focused analysis of time allocation patterns associated with individuals at lower literacy levels.

### Background questionnaire

The background questionnaire included several demographic items. For this project, we used data on age, native English speaker status, self-reported learning disability status, employment status, race/ethnicity, and educational attainment. Age was categorized into “younger,” “middle,” and “older” groups. The “younger” group consisted of individuals aged 24 and younger and those aged 25 to 34, the “middle” group included respondents aged 35 to 44, and the “older” group encompassed those aged 45 to 54 and 55 and above.

The background questionnaire also contained several items pertaining to engagement in ICT, reading, writing, and numeracy skills-use at home and at work. Scaling indices of the skills-use variables have been derived with item response theory modeling on the related categorical variables and are available to be used (He et al., 2021). We used the numeric skills-use index variables to investigate associations between time-related variables and respondents’ skills-use at home and at work.

Many of the adults who were assigned Testlet L11 and achieved a Level 2 and below literacy level (*N* = 359) did not respond to the skills-use at work items (51, 22, 36, and 35% missing data for ICT, reading, writing, and numeracy skills at work, respectively). Therefore, in research question 3, we included the at home, but not the at work skills-use variables.

## Analytic approach

Initially, we described the demographic and skills-use variables across each literacy proficiency level (Below Level 1, Level 1, 2, 3, 4, and 5). Means were computed for the skills-use variables, while frequency distributions were generated for the demographic variables.

To address our first research question, we conducted a descriptive analysis of Testlets L11 through L13. We specifically examined the mean time allocated to each of the nine items across the three timing variables (time to first action, time for last action, and total time) by literacy proficiency level. We focused on comparing the timing among individuals categorized as Below Level 1, Level 1, and Level 2 against those at Levels 3 and above. Furthermore, we descriptively explored and visually represented these trends for Testlets L12 and L13 to discern their consistency among individuals scoring at Level 2 or below on the more challenging items.

Furthermore, we conducted one-way ANOVA significance tests for Testlets L11 through L13. These tests assessed potential differences in time allocation on the nine items across literacy levels, considering the three timing variables. Additionally, we performed one-way ANOVAs across all testlets to investigate total mean differences on the nine items by literacy proficiency level for each of the three timing variables.

To address our second research question, we conducted a K-means cluster analysis to explore distinct patterns of time allocation among adult participants in Testlet L11 who scored Level 2 or below on the PIAAC literacy scale. We employed the ‘kmeans’ clustering function from the stats package, a core package offered in R Studio (R Core Team, 2021), to investigate clustering of the nine items across time to first action, time for last action, and total time spent. In total, we examined 27 different variables. For clarity and ease of interpretation, we converted these timing variables from milliseconds to seconds.

To gauge the effectiveness of the clustering, we calculated silhouette scores using the cluster package in R Studio (Maechler et al., 2021), for a range of cluster numbers spanning from 2 to 8. Silhouette scores, ranging from −1 to 1, serve as a metric to assess the clarity and separation of clusters. Furthermore, the conceptual evaluation of these clusters was based on the timing results from the first research question.

To address the third research question, we used regression in R Studio to investigate the relation between essential individual and contextual factors to the number of clusters that emerged from the data in research question 2. Depending on the number of clusters identified in the second research question, we anticipated employing the ‘glm’ function from the stats package in R Studio to conduct logistic regression for two clusters or multinomial logistic regression for more than two clusters. Predictors of cluster membership included total literacy scale score, age, native English speaker status, self-reported learning disability, employment status, race/ethnicity, educational attainment, and skills-use at home (ICT, reading, writing, and numeracy).

## Results

### Preliminary analyses

Table 1 presents mean skills-use (ICT, reading, writing, and numeracy) across the literacy levels for all testlets and solely for Testlet L11. Table 2 includes demographic frequencies across the literacy levels for all testlets and solely for Testlet L11. Below Level 1 and Level 5 groups consisted of substantially smaller sample sizes (*Ns* = 24 and 29, respectively); thus, we combined the Below Level 1 with Level 1 groups and the Level 4 with Level 5 groups. Across all testlets, respondents in the lower-scoring literacy groups (Levels 1 and below and Level 2) reported lower skills-use across ICT at home, and all reading, writing, and numeracy domains compared to the Level 3 and Levels 4 and 5 groups. Additionally, respondents in the lower literacy groups reported lower educational attainment than Level 3 and Levels 4 and 5. Respondents in the lowest literacy groups (Levels 1 and below) more frequently reported being in the oldest group, self-identifying as Black, and self-identifying as Hispanic. Respondents in the higher literacy groups reported the highest frequencies of being native English speakers and being employed. Similar patterns were observed for Testlet L11.

**Table 1:** Means and standard deviations of skills-use across literacy levels.

Variable	Below Level 1 & Level 1		Level 2		Level 3		Levels 4 & 5	
	*M*(*SD*)	Min–Max	*M*(*SD*)	Min–Max	*M*(*SD*)	Min–Max	*M*(*SD*)	Min–Max
**All Testlets**								
ICT at Home	1.81(1.12)	−0.77 – 6.40	2.00(0.99)	−0.79 – 7.71	2.30(0.85)	−0.79 – 6.40	2.66(0.74)	0.27 – 5.05
ICT at Work	1.14(1.14)	−0.25 – 5.46	1.11(1.11)	0.01 – 5.46	1.06(1.06)	0.01 – 5.46	1.06(1.06)	0.25 – 5.46
Reading at Home	2.35(1.14)	−1.30 – 7.43	2.49(0.94)	−0.77 – 7.43	2.66(0.82)	−0.77 – 7.43	2.82(0.73)	0.69 – 7.43
Reading at Work	2.12(1.12)	−0.16 – 7.02	2.11(1.05)	−0.71 – 7.02	2.29(0.90)	−0.96 – 7.02	2.53(0.77)	−0.56 – 5.05
Writing at Home	2.07(1.19)	−0.30 – 6.10	2.19(1.05)	−0.30 – 6.10	2.33(0.91)	−0.30 – 6.10	2.59(0.73)	−0.30 – 6.10
Writing at Work	2.22(1.19)	0.06 – 5.80	2.14(1.05)	0.06 – 5.80	2.28(0.91)	0.06 – 5.80	2.39(0.73)	0.06 – 5.02
Numeracy at Home	2.12(0.90)	−0.51 – 4.14	2.28(0.93)	−0.51 – 6.17	2.45(0.88)	−0.51 – 6.17	2.71(0.74)	0.14 – 6.17
Numeracy at Work	2.11(1.10)	−0.09 – 6.05	2.14(0.99)	−0.09 – 6.05	2.27(0.97)	−0.09 – 6.05	2.47(1.00)	−0.09 – 6.05
**Testlet L11**								
ICT at Home	1.78(1.16)	−0.78 – 6.40	2.05(1.00)	−0.47 – 5.62	2.29(0.86)	−0.79 – 5.09	2.66(0.82)	0.31 – 4.63
ICT at Work	1.86(1.09)	0.25 – 4.02	1.90(1.10)	0.25 – 5.46	2.19(1.04)	0.47 – 5.46	2.48(1.08)	0.43 – 5.46
Reading at Home	2.32(0.92)	−0.26 – 4.69	2.49(0.98)	−0.77 – 7.43	2.70(0.84)	−0.62 – 7.43	2.76(0.69)	0.69 – 6.04
Reading at Work	2.08(1.13)	−0.13 – 7.02	2.12(1.00)	−0.71 – 7.02	2.22(0.82)	−0.96 – 4.47	2.47(0.83)	−0.56 – 4.51
Writing at Home	2.12(1.24)	−0.30 – 5.03	2.23(1.04)	−0.30 – 4.95	2.39(0.90)	−0.30 – 6.10	2.56(0.80)	−0.30 – 4.62
Writing at Work	2.26(1.14)	−0.06 – 4.84	2.21(1.16)	0.06 – 5.80	2.26(0.97)	0.06 – 5.80	2.37(0.93)	0.06 – 4.56
Numeracy at Home	2.17(0.84)	0.14 – 3.61	2.31(0.95)	0.14 – 6.17	2.49(0.90)	−0.51 – 6.17	2.72(0.80)	0.17 – 5.60
Numeracy at Work	2.20(1.00)	0.65 – 6.05	2.13(0.99)	0.07 – 6.05	2.26(0.98)	0.12 – 6.05	2.31(1.14)	−0.09 – 6.05

*M*, mean; *SD*, standard deviation; Min–Max, minimum–maximum.

**Table 2:** Frequencies of demographic data across literacy levels.

	Below Level 1 & Level 1				Level 2				Level 3				Levels 4 & 5			
	All Testlets		Testlet L11		All Testlets		Testlet L11		All Testlets		Testlet L11		All Testlets		Testlet L11	
	*N*	%	*N*	%	*N*	%	*N*	%	*N*	%	*N*	%	*N*	%	*N*	%
Learning Disability	259	15%	96	15%	858	8%	264	8%	1,141	6%	355	4%	450	4%	128	4%
Education																
Less than High School	257	25%	96	27%	856	11%	264	13%	1,138	5%	355	6%	449	2%	127	4%
High School	257	54%	96	54%	856	50%	264	51%	1,138	35%	355	34%	449	18%	127	20%
More than High School	257	21%	96	19%	856	31%	264	37%	1,138	60%	355	60%	449	80%	127	76%
Age																
Younger	259	39%	96	38%	858	44%	264	50%	1,142	39%	355	44%	450	42%	128	41%
Middle	259	17%	96	21%	858	18%	264	16%	1,142	20%	355	18%	450	23%	128	23%
Older	259	44%	96	42%	858	38%	264	34%	1,142	41%	355	38%	450	35%	128	35%
Employment																
Employed	259	66%	96	61%	858	71%	264	74%	1,142	80%	355	78%	450	83%	128	83%
Unemployed	259	15%	96	14%	858	10%	264	11%	1,142	6%	355	7%	450	4%	128	4%
Out of Labor Force	259	19%	96	25%	858	19%	264	15%	1,142	14%	355	15%	450	13%	128	13%
Native Speaker	259	79%	96	75%	858	81%	263	80%	1,142	91%	355	90%	450	92%	128	90%
Hispanic	259	22%	96	24%	857	14%	263	19%	1,139	6%	355	5%	450	4%	128	7%
Race																
White	259	47%	96	56%	857	60%	263	74%	1,139	78%	355	82%	450	85%	128	89%
Black	259	24%	96	34%	857	16%	263	20%	1,139	9%	355	11%	450	4%	128	4%
Asian	259	5%	96	10%	857	6%	263	6%	1,139	5%	355	7%	450	5%	128	7%

*N*, number of respondents with complete data. Percentages indicate the proportion of individuals in each demographic category relative to the total number of individuals (*N*) in each literacy level.

### Time allocation patterns across adults with lower compared to higher literacy skills

**Figure 2:**
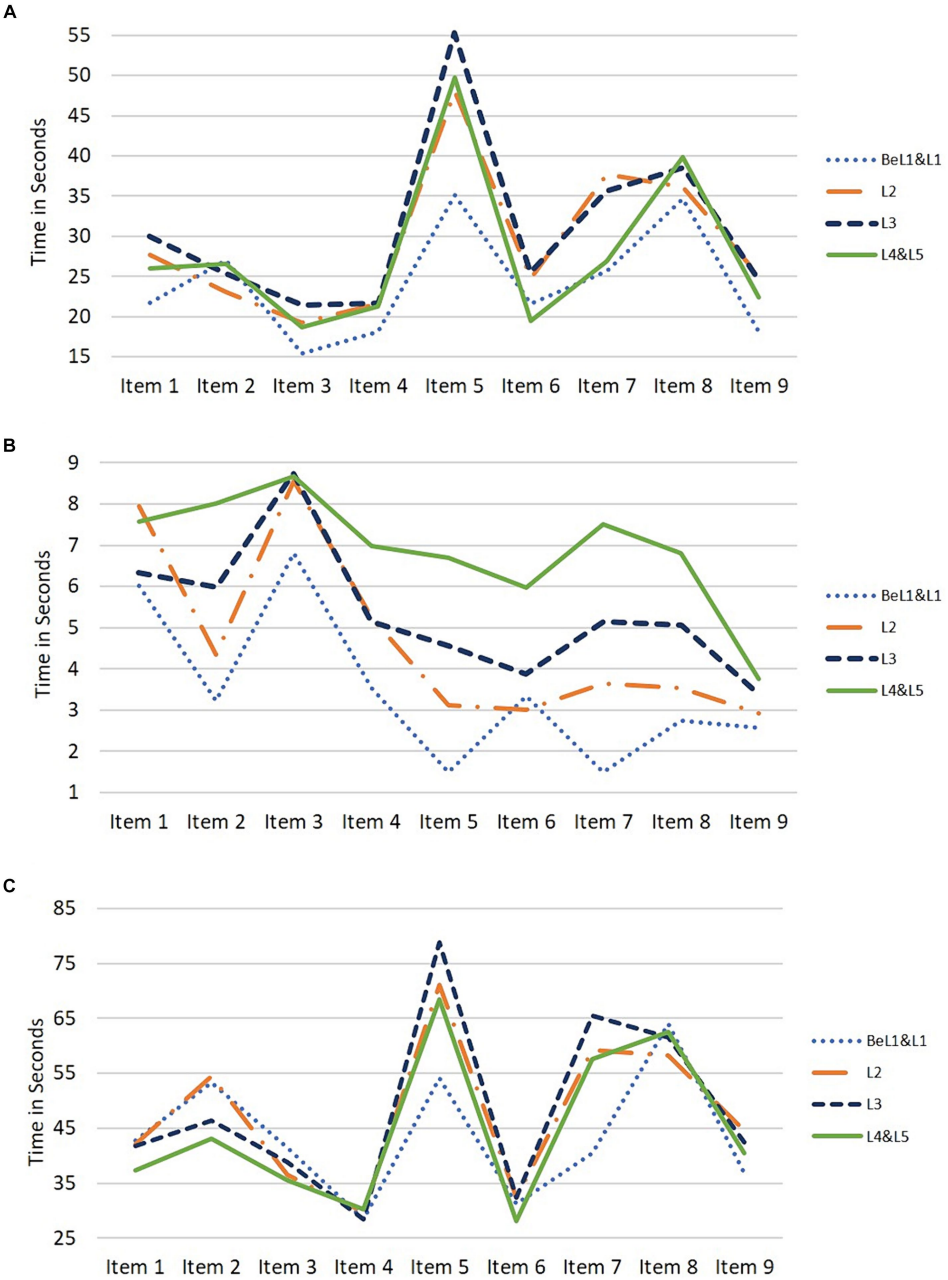
Timing variables by literacy proficiency group. **(A)** Mean time to first action. **(B)** Mean time for last action. **(C)** Mean total time.

To address the first research question, we first examined patterns of time allocation in Testlets L11 through L13 on each of the nine items separately for each timing variable (time to first action, time for last action, total time) by literacy proficiency level (Levels 1 and below, Level 2, Level 3, Levels 4/5). These findings are visualized in Figure 2 for Testlet L11. Generally, respondents scoring at Level 1 and Below Level 1 on the PIAAC literacy domain allocated less time to items across all three timing variables compared to those scoring at Level 2 or above. Respondents at Level 2 showed similar time allocation patterns for time to first action and total time as Levels 3 and above; however, they seemed to spend less on time for the last action than the higher levels.

The time allocation patterns for the nine items, categorized by literacy level, were also visualized for respondents assigned to Testlets L12 and L13 (refer to Supplementary Figures S1, S2). Across the three timing variables, those scoring at Level 1 and Below Level 1 on Testlets L12 and L13 seemed to allocate the least amount of time on more items compared to Testlet L11. Similarly, respondents scoring at Level 2 on these testlets also appeared to allocate less time than the higher literacy levels on more items compared to Testlet L11.

Next, we investigated potential significant differences in the timing variables across the literacy proficiency levels for each of the nine items in Testlet L11. Supplementary Table S4 presents ANOVA results along with pairwise comparisons, while Supplementary Table S5 displays the mean time spent in seconds for each item across the different literacy levels. Respondents scoring at Levels 1 and below demonstrated significantly shorter time to first action on items three, five, seven, and nine compared to those with higher literacy scores (*ps* < 0.05). Respondents scoring at Level 2 spent more time on item seven than those at Levels 4 and 5 (*p* < 0.01). In terms of time for the last action, respondents at Levels 1 and below spent significantly less time on items two, four, five, six, seven, and eight compared to individuals with higher literacy scores (*ps* < 0.05). Respondents at Level 2 also exhibited shorter time for last action than higher-scoring respondents on items two, five, six, seven, and eight (*ps* < 0.05). Overall, regarding total time spent, respondents at Levels 1 and below spent substantially less time than those with higher scores on items 5 and 7; however, no differences were noted for respondents at Level 2 compared to those who achieved a higher literacy level.

Significance tests were also conducted across the literacy levels for each of the nine items in Testlets L12 and L13. ANOVA results with pairwise comparisons for Testlets L12 and L13 can be found in Supplementary Tables S6, S7. In both testlets across the three timing variables, respondents who scored at Levels 1 and below demonstrated the least amount of time on more items compared to Testlet L11. Similarly, those who scored at Level 2 in Testlets L12 and L13 also allocated less time than the higher literacy levels on more items compared to respondents in Testlet L11. These findings suggest that Testlets L12 and L13 posed greater difficulty for respondents at lower literacy levels than Testlet L11.

Finally, we calculated the total average across timing variables for all three testlets and conducted one-way ANOVAs to ascertain differences by literacy level. In Testlet L11, individuals at Levels 1 and below allocated significantly less time to first action compared to Levels 2 and 3 (*ps* < 0.01 and 0.001, respectively). No significant differences were observed between Level 2 and higher-scoring respondents. Regarding time for the last action, respondents scoring Levels 1 and below allotted significantly less time than Levels 2 and above (*ps* < 0.001). Those at Level 2 spent significantly less time than Levels 4 and 5 (*p* < 0.001). However, for total time spent, no significant differences were noted between Levels 2 or below and the higher literacy levels. Descriptive statistics for the total average across timing variables for Testlet L11 are presented in Table 3. Supplementary Table S8 contains ANOVA results with pairwise comparisons for Testlets L11 through L13.

**Table 3:** Means and standard deviations of timing variables across literacy proficiency groups for Testlet L11.

	Time to first action				Time for last action				Total time			
	Mean	*SD*	Bottom 5%	Top 5%	Mean	*SD*	Bottom 5%	Top 5%	Mean	*SD*	Bottom 5%	Top 5%
Total	29.13	15.46	11.36	56.94	5.17	3.28	1.63	11.66	47.08	26.50	20.74	88.36
Below Level 1 & Level 1	24.13	20.33	1.63	56.77	3.47	2.76	0.17	8.89	43.60	29.82	5.15	101.96
Level 2	29.24	15.74	10.27	57.08	4.70	3.24	1.61	10.53	47.51	34.13	19.60	87.30
Level 3	30.87	14.93	14.94	59.35	5.36	2.93	2.38	11.37	48.45	21.37	25.62	89.24
Levels 4 & 5	27.85	10.55	15.36	49.30	6.88	3.76	2.79	14.94	44.80	16.85	24.70	81.06

*SD*, standard deviation; Bottom and Top 5%, average timing of respondents in the 5th and 95th percentile of the distribution.

Supplementary Table S9 contains the descriptive statistics for Testlets L12 and L13 across literacy proficiency groups. Participants achieving Levels 1 and below in Testlets L12 and L13 displayed significantly shorter total time than respondents in the higher literacy levels; however, this result was not observed in Testlet L11. This finding reflects the item difficulty of Testlets L12 and L13, suggesting that individuals scoring Below Level 1 through Level 2 allocated less time than the higher literacy levels on more of the items as the difficulty of the testlets increased. Consequently, we opted to replicate the cluster analysis using Testlet L11 exclusively, as it contained the easiest items, which are the most appropriate for individuals who scored at the lower literacy levels (Levels and below).

### Clusters of adults with lower skills by time allocation

To address the second research question, we used *K*-means cluster analysis based on all time-related variables (nine items × three timing variables = 27 variables) to examine whether there were distinct groups of respondents with lower literacy skills (at or below Level 2) on Testlet L11. We initially selected *K* = 2 clusters, supported by the findings in research question one, which indicated that adults at Level 2 spent more time on the task than Level 1 and Below Level 1. We thus anticipated that there would be at least 2 clusters of adults based on their time spent. The silhouette score analysis across the range of 2 through 8 clusters indicated that the highest silhouette score was observed for *K* = 2 clusters, validating that this solution was the best fit to our data (see Supplementary Figure S3). Therefore, we selected 2 clusters as the optimum number of clusters.

**Figure 3:**
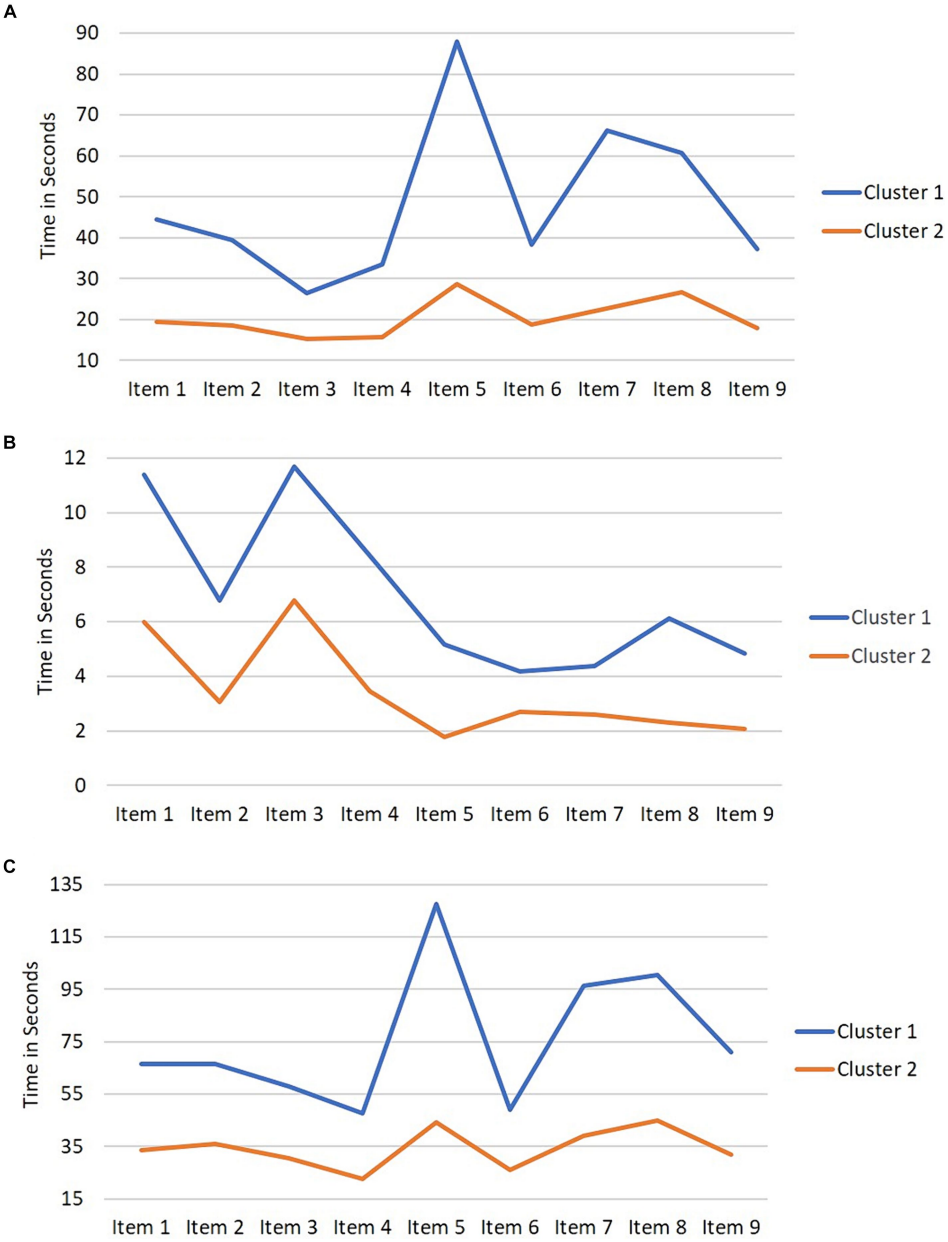
Timing variables by cluster. **(A)** Mean time to first action. **(B)** Mean time for last action. **(C)** Mean total time.

The visual representation of the 2-cluster solution is depicted in Figure 3. The findings indicate that Cluster 1 allocated more time than Cluster 2 across all nine items for the time to first action, time for last action, and total time variables. Additionally, we conducted *t*-tests to determine the significance of these differences across the nine items. Cluster 1 spent significantly more time than Cluster 2 on all nine items across all three timing variables (*ps* < 0.001). Supplementary Table S10 includes descriptive statistics for time spent in seconds on each item by cluster.

Next, we conducted one-way ANOVAs to compare mean differences between Clusters 1 and 2 across the average of the nine items for time to first action, time for last action, and total time. The mean and standard deviation (*M* [*SD*]) for time to first action, time for last action, and total time was 48.19 (18.06), 7.00 (3.60), and 75.90 (22.14) seconds, respectively, for Cluster 1 and 20.39 (8.85), 3.42 (2.36), and 34.22 (13.23) seconds, respectively, for Cluster 2. There were statistically significant differences between the means of the two clusters for time to first action (*F*(1, 357) = 377, *p* < 0.001), time for last action (*F*(1, 357) = 119.7, *p* < 0.001), and total time (*F*(1, 357) = 472, *p* < 0.001), indicating that respondents in Cluster 1 spent notably more time on the items than respondents in Cluster 2.

### Predictors of cluster membership

To address the third research question, logistic regression was employed to investigate whether various individual factors (such as literacy scores, educational attainment, and presence of a learning disability) along with contextual factors (ICT, reading, writing, and numeracy activities at home) predicted the clusters derived from respondents’ timing behaviors as explored in research question two. Table 4 reports the means of the skills-use and literacy scores and Table 5 contains demographic frequencies across Clusters 1 and 2. Table 6 reports correlations between the continuous variables (literacy score with ICT, reading, writing, and numeracy skills-use at home).

**Table 4:** Means and standard deviations of skills-use and literacy scores across cluster membership.

Variable	Cluster 1				Cluster 2			
	*N*	Mean	*SD*	Min–Max	*N*	Mean	*SD*	Min–Max
**Skills-Use**								
ICT at Home	80	1.89	0.91	0.05–3.69	218	2.03	1.09	−0.78–6.50
Reading at Home	96	2.53	0.77	0.86–4.69	263	2.41	1.03	−0.77–7.42
Writing at Home	86	2.37	1.09	−0.30 –5.03	236	2.14	1.09	−0.30–4.95
Numeracy at Home	92	2.28	0.93	0.17–5.28	253	2.27	0.92	0.14–6.17
Literacy Score	96	248.90	24.29	181.65–299.54	263	238.94	29.04	150.38–298.12

*N*, number of respondents in the entire cluster with complete data. *SD*, standard deviation; Min–Max, minimum–maximum.

**Table 5:** Demographic frequencies across cluster membership.

Variable	Cluster 1		Cluster 2	
	Frequency	%	Frequency	%
Total	96		263	
Learning Disability	10	10%	26	10%
Education				
Less than High School	15	16%	44	17%
High School	44	46%	141	54%
More High School	37	39%	78	30%
Age				
Younger	25	26%	143	54%
Middle	15	16%	47	18%
Older	56	58%	73	28%
Employment				
Employed	63	66%	191	73%
Unemployed	14	15%	27	10%
Out of Labor Force	19	20%	45	17%
Native Speaker	74	77%	209	79%
Race				
White	48	50%	146	56%
Hispanic	16	17%	56	21%
Black	24	25%	40	15%
Asian	8	8%	12	5%

Percentages indicate the proportion of individuals in each demographic category relative to the total number of individuals in each cluster.

**Table 6:** Correlations between continuous predictors.

	1	2	3	4	5
Literacy Score	–	0.10	0.12*	0.10	0.11*
ICT at Home	0.08	–	0.46***	0.50***	0.40***
Reading at Home	0.07	0.48***	–	0.46***	0.51***
Writing at Home	0.09	0.50***	0.45***	–	0.45***
Numeracy at Home	0.10	0.40***	0.47***	0.45***	–

Correlations at the bottom of the diagonal were estimated using listwise deletion. Correlations at the top of the diagonal were estimated using pairwise deletion. ***Correlation is significant at <.001, **Correlation is significant at .01, and *Correlation is significant at .05.

Initial analyses were conducted to evaluate missing data across all variables. The majority of missing data was observed for the skills-use variables at home; among 359 respondents, 17, 10, and 4% did not provide responses for ICT, writing, and numeracy skills at home, respectively. To assess whether the missingness followed a Missing at Random (MAR) pattern, a logistic regression was performed using the literacy and demographic variables in research question three to predict missingness on the skills-use at home variables. The demographic frequencies of respondents with missing and complete skills-use data are reported in Supplementary Table S11. The mean and standard deviation of the literacy domain scores for individuals with missing and complete skills-use data are 231.99 (32.26) and 244.68 (26.04), respectively. The parameter estimates of the model predicting missingness are reported in Supplementary Table S12, which displays the estimated coefficients, odds ratios (OR), 95% confidence intervals (95% CI), and *p*-values.

According to the findings, respondents with higher literacy scores were significantly less likely to have missing data on the skills-use variables at home (OR = 0.58, 95% CI = 0.50–0.81, *p* < 0.001). Furthermore, individuals who reported being unemployed were significantly less likely to exhibit missing data on the skills-use variables at home compared to those who were employed (OR = 0.17, 95% CI = 0.05–0.44, *p* < 0.001). These results suggest that missing data for skills-use at home is MAR; thus, we handled the missing data using multiple imputation with the mice and miceadds packages in R studio (van Buuren and Groothuis-Oudshoorn, 2011; Robitzsch and Grund, 2023). Additionally, results from complete case analysis (i.e., listwise deletion) were also reported to evaluate whether the findings remained consistent when cases with missing data were excluded. Supplementary Table S13 presents descriptive statistics for individuals with complete data, including average skills-use and literacy scores, categorized by cluster membership. Supplementary Table S14 provides demographic frequency data categorized by cluster membership.

Table 7 displays the estimated coefficients, ORs, 95% CIs, and *p*-values derived from the logistic regression analysis involving all 359 respondents. Writing skills-use at home approached significance, indicating that higher utilization of writing skills at home was associated with lower odds of belonging to Cluster 2 (OR = 0.68, 95% CI = 0.46–1.01, *p* = 0.054). Furthermore, scores on the literacy domain assessment were significantly linked with reduced odds of being in Cluster 2 (OR = 0.43, 95% CI = 0.30–0.62, *p* < 0.001). Respondents reporting a learning disability were also associated with decreased odds of Cluster 2 membership (OR = 0.28, 95% CI = 0.08–0.90, *p* = 0.033). Age exhibited a significant negative correlation with Cluster 2 membership; middle-aged and older individuals demonstrated significantly lower odds of belonging to Cluster 2 compared to younger individuals (OR = 0.31, 95% CI = 0.13–0.77, *p* = 0.012, and OR = 0.08, 95% CI = 0.04–0.19, *p* < 0.001, for middle-aged and older individuals, respectively). Moreover, individuals who were unemployed (OR = 0.41, 95% CI = 0.17–0.98, *p* = 0.046) and those who identified as Black (OR = 0.18, 95% CI = 0.08–0.43, *p* < 0.001) had significantly lower odds of belonging to Cluster 2 compared to employed individuals and White respondents, respectively.

**Table 7:** Individual and contextual factors predicting cluster membership.

	Logit	Odds Ratio	95% CI	*p*-value
(Intercept)	2.77	15.96	3.56–71.56	<.001
Skills-Use				
ICT Home	0.32	1.37	0.91–2.07	.133
Reading at Home	0.13	1.13	0.73–1.75	.573
Writing at Home	−0.38	0.68	0.46–1.01	.054
Numeracy at Home	−0.16	0.85	0.58–1.24	.399
Literacy Score	−0.84	0.43	0.30–0.62	.000
Learning Disability	−1.29	0.28	0.08–0.90	.033
Education^a^				
High school	0.38	1.46	0.59–3.57	.412
More than high school	0.31	1.36	0.50–3.66	.543
Age^b^				
Middle age	−1.17	0.31	0.13–0.77	.012
Older age	−2.48	0.08	0.04–0.19	<.001
Employment^c^				
Not Employed	−0.89	0.41	0.17–0.98	.046
Out of the labor force	−0.69	0.50	0.23–1.10	.086
Native Speaker	0.33	0.39	0.48–4.04	.549
Race and Ethnicity^d^				
Hispanic	−0.70	0.50	0.15–1.64	.249
Black	−1.69	0.18	0.08–0.43	<.001
Asian	−1.27	0.28	0.06–1.34	.112

*N* = 359; Cluster 1 is the reference category. 95% CI, 95% confidence interval.

a Less than high school is the comparison.

b Younger age is the comparison.

c Employed is the comparison.

d White is the comparison.

Supplementary Table S15 contains the parameter estimates from the complete case analysis (*N* = 272 participants with complete data). Findings from the logistic regression utilizing complete case analysis to address missing data were largely similar to those from the analysis using multiple imputation, with two notable exceptions. Specifically, the utilization of writing skills at home emerged as a significant predictor of cluster membership in the complete case analysis (OR = 0.58, 95% CI = 0.36–0.91, *p* = 0.020); however, writing skills-use was not significant after including all 359 participants and imputing the missing data (OR = 0.68, 95% CI = 0.46–1.01, *p* = 0.054). In the model with 272 participants with complete data, the association with learning disability approached significance (OR = 0.29, 95% CI = 0.08–1.13, *p* = 0.065), yet was significant in the model with imputed data (OR = 0.28, 95% CI = 0.08–0.90, *p* = 0.033). Nevertheless, the direction and magnitude of the effects from the complete case analysis remained highly consistent with the model involving 359 participants that employed multiple imputation methods to handle missing data. Discrepancies in significance values may be attributed to the reduced sample size in the complete case analysis model.

## Discussion

The purpose of the current study was threefold. First, we explored time allocation patterns (time to first action, time for last action, total time) of adults across all levels of proficiency on the PIAAC digital literacy items. Second, we examined whether we could identify different time allocation patterns (i.e., clusters) amongst adults who demonstrated lower literacy levels (Levels 2 and below). Finally, we examined whether individual and contextual factors predicted cluster membership with this group of adults. Results suggested that adults with the lowest literacy levels (Level 1 and Below Level 1) spent the least amount of time across many of the nine literacy items on time to first action and time for last action than adults who achieved Level 2 and higher (Levels 3 and above). Within the group of respondents with lower literacy skills (Levels 2 and below), we identified two distinct clusters, such that respondents in Cluster 1 spent significantly more time on the digital literacy items than respondents in Cluster 2. Finally, we found that adults with lower literacy skills who reported having a learning disability, being older, unemployed, identifying as Black, and having relatively higher literacy scores had a higher probability of Cluster 1 membership (spending more time). Engagement in writing activities approached significance (*p* = 0.054), suggesting that individuals who reported high frequency in writing at home were more likely to be members of Cluster 1. These findings have educational implications for adults with lower literacy skills as well as implications for future research with this population.

### Time allocation patterns

Across all testlets, adults with higher literacy levels (Levels 3 and 4/5) demonstrated an overall pattern of spending more time across the items than adults with lower literacy levels (Level 2 and Levels 1 and below). This is consistent with evidence from work with proficient adults, which suggests that time spent on digital assessments is related to better performance (Liao et al., 2019). Similarly, some emerging work with adults with lower literacy skills suggests that more time spent on digital tasks may indicate better performance for some learners (e.g., Fang et al., 2018).

Interestingly, time allocation patterns varied for adults who demonstrated lower literacy skills (Levels 2 and below). Specifically, respondents in Testlet L11 with a Level 2 proficiency score spent overall less time for the last action than those in Levels 3–5. However, they demonstrated similar allocation patterns on time to first action as those in the higher-level proficiency levels on eight of the nine items. On item 7, those who scored at Level 2 spent more time to first action than Levels 3 and above. Adults at Levels 1 or below on average demonstrated patterns of spending the least amount of time than Levels 2 and above on many of the items across time to first action and time for last action. These patterns seem to also be echoed in our finding that two distinct clusters emerged within the group of respondents who scored at Levels 2 and below, and that the lowest literacy scores were associated with less time spent interacting with the items. These findings raise questions as to the challenges faced by respondents with lower skills when interacting with digital test items. For example, it is possible that respondents with lower literacy skills become fatigued over time and/or are unable to manage the cognitive (i.e., reading) and/or technology demands within the digital environment. Future research should explore the nature of the challenges that adults with lower skills face while interacting with digital assessments, as this may inform testing strategies and reading skills that could be taught in adult education programs to increase students’ performance.

### Individual and contextual predictors

Generally, individual differences and contextual factors were similar across the two clusters, with the exceptions of age, race, literacy, and writing skills at home. In particular, younger adults (ages <24–34) were more likely to be in Cluster 2 (overall less time spent). Although the higher literacy levels (3 through 5) appear to have a higher representation of respondents who are younger and have higher ICT skills (see Tables 1, 2); our results focusing on Levels 2 and below emphasize that younger adults facing literacy challenges may not always demonstrate adequate engagement on digital literacy assessments. This finding aligns with Fang et al. (2018), who found four clusters, or groups, of adults based on timing actions and accuracy in a digital reading educational program. One of the clusters included a group of under-engaged readers that were fast, but relatively inaccurate on the digital tasks. The authors did not report information regarding individual and contextual factors (e.g., age, race, educational attainment, skills-use) of each profile; however, based on our findings, it may be the case that younger adults with lower literacy skills tend to be under-engaged readers.

Adults with lower literacy skills, particularly those who are older, self-identify as Black, report having a learning disability, demonstrate relatively higher literacy performance, and engage in greater writing activities at home, show a higher likelihood of belonging to Cluster 1 and spending more time on tasks. This aligns with previous research indicating that individuals with learning difficulties (e.g., Ziegler et al., 2006) and older adults (e.g., Comings et al., 1999; Sabatini et al., 2011; Greenberg et al., 2013) tend to exhibit better attendance in adult literacy intervention studies and educational classes. Moreover, daily exposure to printed materials among adult literacy students correlates with increased persistence and engagement in adult education (Greenberg et al., 2013), which aligns with our findings highlighting a connection between frequent engagement in writing activities at home and extended time spent in the digital literacy assessment. Additionally, recent research suggests that adults with low literacy skills who identify as Black tend to demonstrate better attendance in adult literacy programs across the state of Georgia compared to those who identify as White (Tighe et al., 2023b). Taken together, our study’s results and prior literature suggest that these groups exhibit higher levels of persistence, albeit being disproportionately represented among adults with lower literacy skills. These findings underscore the need for further research to better understand the factors and barriers affecting the performance of these groups of adults on digital literacy assessments. Such insights could inform tailored instructional strategies for these specific groups, potentially leading to improved outcomes on digital literacy assessments.

## Educational implications

There are few educational implications that can be made from the results of this study. First, adults with lower literacy skills, particularly those at Levels 1 and below, spent considerably less time on the items, suggesting a potential need for support in sustaining their engagement during interactions with digital literacy assessments. Second, within the group scoring Levels 2 and below, individuals in Cluster 1 spent more time on digital literacy items and were more likely to score higher than those in Cluster 2, who spent much less time. However, Cluster 1, on average, spent more time (*M* = 75.90 s) than respondents with higher literacy proficiency levels (Levels 3 and above; *Ms* = 44.80–48.50 s). A similar finding by Fang et al. (2018) identified a group of adults spending more time on digital literacy tasks but struggling with accuracy compared to those spending less time. Therefore, sustained engagement may benefit adults with lower literacy skills, but its impact has limits. These results suggest that respondents may require additional support with foundational literacy skills to either enhance sustained engagement (for respondents in Cluster 2 who spent much less time) or to become better, more efficient readers (as seen in respondents in Cluster 1).

To date, a few intervention studies have focused on targeting the foundational reading skills of adults with lower literacy skills (e.g., Alamprese et al., 2011; Greenberg et al., 2011; Hock and Mellard, 2011; Sabatini et al., 2011). However, many of these studies reported minimal to no gains in learners (e.g., Greenberg et al., 2011; Sabatini et al., 2011). More recently, intervention work has explored a digital learning tool called AutoTutor to teach comprehension strategies to adults with lower literacy skills (Graesser et al., 2021; for an example, see www.arcweb.us). Recent evidence suggests that this tool may be a valuable supplement to traditional instruction (Hollander et al., 2021), potentially leading to improvements in the foundational reading skills needed (e.g., decoding, vocabulary) to perform better on literacy assessments, particularly digital literacy assessments.

Furthermore, respondents in the current study who scored at Levels 2 or below, particularly those at Levels 1 and below, might have displayed diminished engagement (or less time) on specific items due to limited use of ICT skills. These indicators of reduced engagement align with the skill profiles linked to Levels 2 and below in the PIAAC literacy domain, highlighting that individuals at these levels often lack skills in interacting with digital literacy texts. Our findings underscore the ongoing importance of exposing adult literacy learners to digital texts. In doing so, there is significant value in prioritizing the development of fundamental ICT skills essential for effective interaction with these texts, including activities such as scrolling, right-clicking, and utilizing web features (Vanek et al., 2020). Integrating technology into adult literacy classrooms has the potential to enhance performance on digital literacy assessments, but careful consideration should be given to the thoughtful integration of technology into classroom instruction (Vanek et al., 2020). Specifically, digital literacy activities should align with academic learning objectives and focus on helping adult literacy learners engage with digital texts relevant to high-stakes test-taking situations (e.g., GED, HiSET).

Moreover, our results from the broader sample of adult respondents suggest that respondents with higher literacy proficiency levels (Levels 3 and above) tend to also have higher engagement in skills-use overall at home and at work (see Table 1). Specific to individuals who scored at Levels 2 and below, respondents who scored higher on the literacy assessment also tended to report higher writing skills-use at home. Previous findings have suggested that literacy instruction in adult education programs that focuses on skills-use can lead to better outcomes (Reder, 2009b, 2019). Thus, instruction that is situated in daily, authentic scenarios that require reading and writing may help adults with lower literacy skills achieve higher gains and become more efficient when taking digital literacy assessments. Specifically, our findings emphasize the importance of adopting a “literacy-in-use” framework, which can help adults with lower literacy skills improve their reading and writing in different types of contexts and situations, some of which may occur in a digital environment, such as reading memos, emails, and interpreting instructions, diagrams, and/or maps (Trawick, 2019). This type of framework transcends instruction that is only focused on the foundational skills of reading (e.g., decoding, vocabulary knowledge), and emphasizes other higher-order skills that are necessary for adults with lower literacy skills (Tighe et al., 2023a,c), including making inferences, relating different pieces of a text or multiple texts to each other, and evaluating the quality of information in texts (Trawick, 2019).

## Limitations and future directions

There were a few limitations to this study. First, this study focused on the digital literacy items, which are part of the computer-based PIAAC assessment. Thus, we were unable to capture the performance of all adults with lower literacy skills who may have opted out of the computer-based assessment or may not have qualified to take the assessment due to limited or no technology use. This suggests that there are adults with lower literacy skills who have virtually no technology experience and would benefit from increased attention to ICT skills in adult education programs, especially because more high-stakes assessments, such as the GED and HiSET, are becoming increasingly digital.

A second limitation of this study stems from the race and ethnicity item in the PIAAC dataset, which introduces conceptual complexities by combining race and ethnicity, notably because identifying as Hispanic denotes an ethnicity rather than a race. Consequently, it is imperative to recognize that our assessment of the race and ethnicity categories in the current study is a less precise interpretation of their effects on cluster membership. For instance, we designated individuals identifying as White as the reference group and generated dummy codes for Black, Hispanic, and Asian categories. This revised interpretation now contrasts individuals who identified as Hispanic (potentially also identifying as White or Black) solely against those identifying as White, non-Hispanic. Ideally, these respondents would be compared against all other non-Hispanic individuals in the sample. Unfortunately, the PIAAC dataset limited respondents’ ability to distinguish ethnicity from race.

A third constraint relates to the observed discrepancy between the logistic regression models employed in research question 3—multiple imputation and complete case analysis—to handle missing data. In our analysis, we found that writing at home significantly predicted cluster membership in the complete case analysis (*p* = 0.020), yet only approached significance post-imputation (*p* = 0.054). Similarly, the learning disability variable approached significance in the complete case analysis (*p* = 0.065), but was statistically significant following imputation with the augmented sample size (*p* = 0.033). The consistent direction and magnitude of effects between the complete case and imputed models suggest that this inconsistency could primarily be attributed to the larger sample size represented in the imputed model. Nevertheless, caution is warranted in the interpretation of findings related to the learning disability and writing at home variables due to this discrepancy.

Finally, this study used the PIAAC public-use process data and therefore, we were constrained to the three timing variables (time to first action, time for last action, and total time) that were available. Timing data is informative to understanding the behaviors of test-takers (Cromley and Azevedo, 2006) and provides important information regarding engagement or efficiency of adults with lower literacy skills on digital literacy tasks (e.g., Fang et al., 2018); however, we were not able to elaborate on any challenges specific to ICT skills needed to navigate a digital literacy assessment (e.g., using a mouse to highlight or click through the items). A future direction of our work will be to use the restricted access PIAAC process data to examine specific literacy items to map certain actions (e.g., highlighting, clicking) and sequences of actions, which will help us better understand the challenges adults with low literacy skills may have on a digital assessment and how these may influence item performance. Some previous work using PIAAC PSTRE items suggests that there are differences in the efficiencies of different sequences of actions to solve the items (e.g., He and von Davier, 2015, 2016). The items that were examined in these studies involved higher-order executive function and problem-solving skills, which included fewer adults with lower literacy skills. Thus, this has not been examined specific to PIAAC literacy items, which would require more basic ICT skills (e.g., sample item in Figure 1) for adults facing literacy challenges.

## Conclusion

This study provides important insights into how adults with literacy challenges perform on digital literacy items. The time allocation patterns revealed a stark differentiation between literacy proficiency levels, and, in particular emphasized the heterogeneous nature of time allocation patterns amongst adults facing challenges on digital literacy assessments (Levels 2 and below). Overall, the findings underscore the need for tailored interventions to support adults with lower literacy skills on digital literacy assessments, recognizing that engagement may vary according to skills-use in everyday life, literacy proficiency, and a diverse set of demographic factors.

## Data availability statement

Publicly available datasets were analyzed in this study. This data can be found at: https://nces.ed.gov/pubsearch/pubsinfo.asp?pubid=2014045REV and https://search.gesis.org/research_data/ZA6712?doi=10.4232/1.12955.

## Ethics statement

Ethical approval was not required for the study involving humans in accordance with the local legislation and institutional requirements. Written informed consent to participate in this study was not required from the participants or the participants’ legal guardians/next of kin in accordance with the national legislation and the institutional requirements.

## Author contributions

GK: Conceptualization, Formal analysis, Methodology, Visualization, Writing – original draft, Writing – review & editing. ET: Conceptualization, Funding acquisition, Supervision, Writing – original draft, Writing – review & editing. QH: Conceptualization, Data curation, Formal analysis, Funding acquisition, Methodology, Supervision, Writing – review & editing.

## Footnotes

^1^In the integrated block design, the 18 unique items were assigned to four blocks: block A1 (4 items), block B1 (5 items), block C1 (4 items), and block D1 (5 items). Each testlet consisted of two blocks: L11 (block A1 and B1), L12 (block B1 and C1), and L13 (block C1 and D1). The block B1 and C1 functioned as linking blocks among the three testlets.

^2^The PIAAC adopted item response theory 2PL and the Generalized Partial Credit Model (GPCM) to calibrate item parameters in literacy, numeracy and problem-solving in technology-rich environments (PSTRE). Each item has two item parameters: difficulty parameter and slope parameter. The difficulty parameter indicates the difficulty level in the item according to the latent ability estimation. The slope parameter indicates the discrimination level in the item to identify how sensitive the item can be used to distinguish between latent trait levels.
