# Supplementary material for: It’s about time! Exploring time allocation patterns of adults with lower literacy skills on a digital assessment
Source: Front Psychol. 2024 Jun 7;15:1338014. doi: 10.3389/fpsyg.2024.1338014 (PMC11191086; doi:10.3389/fpsyg.2024.1338014)
Supplement: Supplementary file 1 [file Table_1.DOCX]

**Table S1**

*Item Characteristics of PIAAC Literacy Items in Stage 1*

| **CBA**  **Sequence** | **Unit Order** | **Item Name** | **Item ID** | **Difficulty** | **Slope** |
| --- | --- | --- | --- | --- | --- |
| CBA-L11: 1 | Unit 1 Question 1/1 | 311 Dutch Women | C311B701 | -1.436 | 0.718 |
| CBA-L11: 2 | Unit 2 Question 1/2 | 321 Internet Poll | C321P001 | -0.329 | 1.041 |
| CBA-L11: 3 | Unit 2 Question 2/2 | 321 Internet Poll | C321P002 | -0.968 | 0.519 |
| CBA-L11: 4 | Unit 3 Question 1/4 | 308 Baltic Stock Market | C308A117 | -0.534 | 1.088 |
| CBA-L11: 5 | Unit 3 Question 2/4 | 308 Baltic Stock Market | C308A119 | 0.614 | 1.285 |
| CBA-L11: 6 | Unit 3 Question 3/4 | 308 Baltic Stock Market | C308A120 | -0.202 | 1.27 |
| CBA-L11: 7 | Unit 3 Question 4/4 | 308 Baltic Stock Market | C308A121 | 0.266 | 1.296 |
| CBA-L11: 8 | Unit4 Question 1/2 | 305 TMN AntiTheft | C305A215 | -0.139 | 1.116 |
| CBA-L11: 9 | Unit 4 Question 2/2 | 305 TMN AntiTheft | C305A218 | 0.764 | 1.077 |
| CBA-L12: 1 | Unit 1 Question 1/1 | 315 Mexican Cities | C315B512 | 0.766 | 0.758 |
| CBA-L12: 2 | Unit 2 Question 1/4 | 308 Baltic Stock Market | C308A117 | -0.534 | 1.088 |
| CBA-L12: 3 | Unit 2 Question 2/4 | 308 Baltic Stock Market | C308A118 | 1.260 | 1.009 |
| CBA-L12: 4 | Unit 2 Question 3/4 | 308 Baltic Stock Market | C308A119 | 0.614 | 1.285 |
| CBA-L12: 5 | Unit 2 Question 4/4 | 308 Baltic Stock Market | C308A121 | 0.266 | 1.296 |
| CBA-L12: 6 | Unit 3 Question 1/2 | 305 TMN AntiTheft | C305A215 | -0.139 | 1.116 |
| CBA-L12: 7 | Unit 3 Question 2/2 | 305 TMN AntiTheft | C305A218 | 0.764 | 1.077 |
| CBA-L12: 8 | Unit 4 Question 1/2 | 304 Contact Employer | C304B710 | 0.476 | 1.722 |
| CBA-L12: 9 | Unit 4 Question 2/2 | 304 Contact Employer | C304B711 | 0.892 | 0.964 |
| CBA-L13: 1 | Unit 1 Question 1/1 | 315 Mexican Cities | C315B512 | 0.766 | 0.758 |
| CBA-L13: 2 | Unit 2 Question 1/2 | 304 Contact Employer | C304B710 | 0.476 | 1.722 |
| CBA-L13: 3 | Unit 2 Question 2/2 | 304 Contact Employer | C304B711 | 0.892 | 0.964 |
| CBA-L13: 4 | Unit 3 Question 1/2 | 308 Baltic Stock Market | C308A116 | 1.743 | 0.735 |
| CBA-L13: 5 | Unit 3 Question 2/2 | 308 Baltic Stock Market | C308A118 | 1.260 | 1.009 |
| CBA-L13: 6 | Unit 4 Question 1/4 | 327 Summer Streets | C327P001 | 0.492 | 0.919 |
| CBA-L13: 7 | Unit 4 Question 2/4 | 327 Summer Streets | C327P002 | 0.632 | 0.897 |
| CBA-L13: 8 | Unit 4 Question 3/4 | 327 Summer Streets | C327P003 | 0.937 | 0.972 |
| CBA-L13: 9 | Unit 4 Question 4/4 | 327 Summer Streets | C327P004 | 1.552 | 1.132 |

*Note*: In PIAAC, the Item Response Theory 2-Parameter Logistic Model (2PL) and the Generalized Partial Credit Model (GPCM) were utilized to estimate item parameters for literacy assessment. Each item is characterized by two parameters: the difficulty parameter and the slope parameter. The difficulty parameter reflects the item's difficulty level based on latent ability estimation, while the slope parameter signifies the item's discrimination level, indicating its sensitivity in distinguishing between different levels of the latent trait.

**Table S2**

| **EdLevel3:** | | **Low** | | **Low** | | **Medium** | | **Medium** | | **High** | |
| --- | --- | --- | --- | --- | --- | --- | --- | --- | --- | --- | --- |
| **Native Speaker:** | | **No** | | **Yes** | | **No** | | **Yes** | | **Both** | |
| **Threshold:** | | I | II | I | II | I | II | I | II | I | II |
| **CBA-Core Stage 2 Score** | 0 | 0.900 | 0.950 | 0.872 | 0.922 | 0.850 | 0.900 | 0.822 | 0.872 | 0.800 | 0.850 |
|  | 1 | 0.738 | 0.945 | 0.710 | 0.917 | 0.688 | 0.895 | 0.660 | 0.867 | 0.638 | 0.845 |
|  | 2 | 0.607 | 0.924 | 0.579 | 0.896 | 0.557 | 0.874 | 0.529 | 0.846 | 0.507 | 0.824 |
|  | 3 | 0.505 | 0.887 | 0.477 | 0.859 | 0.455 | 0.837 | 0.427 | 0.809 | 0.405 | 0.787 |
|  | 4 | 0.433 | 0.834 | 0.405 | 0.806 | 0.383 | 0.784 | 0.355 | 0.756 | 0.333 | 0.734 |
|  | 5 | 0.392 | 0.765 | 0.364 | 0.737 | 0.342 | 0.715 | 0.314 | 0.687 | 0.292 | 0.665 |
|  | 6 | 0.380 | 0.680 | 0.352 | 0.652 | 0.330 | 0.630 | 0.302 | 0.602 | 0.280 | 0.580 |

*An Example of the Matrix Design for Literacy Stage 1 Testlet Selection*

*Note*: Probabilities were computed for respondents based on their education level, native speaker status, and CBA-Core Stage 2 scores, with thresholds set at .738 and .945. Respondents below .738 were assigned to Testlet L11; those equal to or greater than .738 but less than .945 were assigned to Testlet L12; and those exceeding .945 were assigned to Testlet L13.

**Table S3**

|  | Testlet L11 | | Testlet L12 | | Testlet L13 | |
| --- | --- | --- | --- | --- | --- | --- |
|  | *Frequency* | % | *Frequency* | % | Frequency | % |
| Total | 843 |  | 887 |  | 967 |  |
| Below Level 1 | 8 | 1% | 14 | 2% | 2 | 2% |
| Level 1 | 88 | 10% | 82 | 9% | 63 | 7% |
| Level 2 | 264 | 31% | 290 | 33% | 300 | 31% |
| Level 3 | 355 | 42% | 357 | 40% | 424 | 44% |
| Level 4 | 120 | 14% | 136 | 15% | 165 | 17% |
| Level 5 | 8 | 1% | 8 | 1% | 13 | 1% |

*Number of Respondents in each testlet by literacy level*

**Table S4**

|  | Time to First Action | | | Time for Last Action | | | Total Time | | |
| --- | --- | --- | --- | --- | --- | --- | --- | --- | --- |
|  | F | *p* | Pairwise t-value | F | *p* | Pairwise t-value | F | *p* | Pairwise t-value |
| Item 1 | .88 | .451 |  | 1.53 | .204 |  | .30 | .824 |  |
| Item 2 | 1.19 | .311 |  | 10.61 | **<.001** |  | .33 | .805 |  |
| BeL1&L1 – L3 |  |  | — |  |  | 3.25** |  |  | — |
| BeL1&L1 – L4&L5 |  |  | — |  |  | 4.80*** |  |  | — |
| L2 – L3 |  |  | — |  |  | 2.73* |  |  | — |
| L2 – L4&L5 |  |  | — |  |  | 4.61*** |  |  | — |
| L3 – L4&L5 |  |  | — |  |  | 2.66* |  |  | — |
| Item 3 | 2.68 | **.046** |  | 3.85 | .05 |  | .77 | .51 |  |
| BeL1&L1 – L3 |  |  | 2.71* |  |  | — |  |  | — |
| Item 4 | 1.25 | .29 |  | 3.53 | **.015** |  | .31 | .817 |  |
| BeL1&L1 – L4&L5 |  |  | — |  |  | 3.21** |  |  | — |
| Item 5 | 7.57 | **<.001** |  | 10.74 | **<.001** |  | 6.24 | **<.001** |  |
| BeL1&L1 - L2 |  |  | 2.87* |  |  | 1.80 |  |  | 2.79* |
| BeL1&L1 – L3 |  |  | 4.65** |  |  | 3.54** |  |  | 4.20*** |
| BeL1&L1 – L4&L5 |  |  | 2.86* |  |  | 5.09*** |  |  | — |
| L2 – L3 |  |  | — |  |  | 2.34 |  |  | — |
| L2 – L4&L5 |  |  | — |  |  | 4.38*** |  |  | — |
| L3 – L4&L5&L5 |  |  | — |  |  | 2.73* |  |  | — |
| Item 6 | 3.25 | **.021** |  | 12.82 | **<.001** |  | 1.19 | .312 |  |
| BeL1&L1 – L4&L5 |  |  | — |  |  | 4.29*** |  |  | — |
| L2 – L4&L5 |  |  | — |  |  | 6.08*** |  |  | — |
| L3 – L4&L5 |  |  | -2.83* |  |  | 4.50*** |  |  | — |

*One-Way ANOVA of Time Spent on Each Item in Testlet L11 by Group*

*Note:* Pairwise comparisons estimated using the Bonferroni correction; ****p*<.001; **<.001; *<.05

**Table S4 (Continued)**

|  | Time to First Action | | | Time for Last Action | | | Total Time | | |
| --- | --- | --- | --- | --- | --- | --- | --- | --- | --- |
|  | F | *p* | Pairwise t-value | F | *p* | Pairwise t-value | F | *p* | Pairwise t-value |
| Item 7 | 6.33 | **<.001** |  | 15.11 | **<.001** |  | 10.78 | **<.001** |  |
| BeL1&L1 - L2 |  |  | 3.32** |  |  | — |  |  | 4.08*** |
| BeL1&L1 – L3 |  |  | 2.84* |  |  | 4.39*** |  |  | 5.64*** |
| BeL1&L1 – L4&L5 |  |  | — |  |  | 6.18*** |  |  | 3.27** |
| L2 – L4&L5 |  |  | -3.29** |  |  | 4.99*** |  |  | — |
| L3 – L4&L5 |  |  | -2.77* |  |  | 3.20** |  |  | — |
| Item 8 | .87 | .458 |  | 12.30 | **<.001** |  | .51 | .678 |  |
| BeL1&L1 – L3 |  |  | — |  |  | 3.37** |  |  | — |
| BeL1&L1 – L4&L5 |  |  | — |  |  | 5.02*** |  |  | — |
| L2 – L3 |  |  | — |  |  | 3.12* |  |  | — |
| L2 – L4&L5 |  |  | — |  |  | 5.06*** |  |  | — |
| L3 – L4&L5 |  |  | — |  |  | 2.82* |  |  | — |
| Item 9 | 3.66 | **.012** |  | 2.32 | .073 |  | 1.61 | .185 |  |
| BeL1&L1 - L2 |  |  | 3.08* |  |  | — |  |  | — |
| BeL1&L1 – L3 |  |  | 2.93* |  |  | — |  |  | — |

*One-Way ANOVA of Time Spent on Each Item in Testlet L11 by Group*

*Note:* Pairwise comparisons estimated using the Bonferroni correction; ****p*<.001; **<.001; *<.05

**Table S5**

|  | Time to First Action | | Time for Last Action | | Total Time | |
| --- | --- | --- | --- | --- | --- | --- |
|  | Mean | *SD* | Mean | *SD* | Mean | *SD* |
| Item 1 |  |  |  |  |  |  |
| BeL1&L1 | 21.66 | 20.20 | 6.02 | 10.00 | 42.68 | 38.15 |
| L2 | 27.65 | 50.49 | 7.95 | 14.75 | 42.12 | 54.86 |
| L3 | 30 | 55.66 | 6.33 | 7.70 | 41.75 | 61.29 |
| L4&L5 | 25.98 | 15.24 | 7.58 | 8.63 | 37.27 | 22.35 |
| Item 2 |  |  |  |  |  |  |
| BeL1&L1 | 26.97 | 27.25 | 3.23 | 7.17 | 53.29 | 49.64 |
| L2 | 23.11 | 20.86 | 4.35 | 6.45 | 54.44 | 225.44 |
| L3 | 25.37 | 15.75 | 5.98 | 7.22 | 46.30 | 33.81 |
| L4&L5 | 26.59 | 30.84 | 8.00 | 9.39 | 43.08 | 36.04 |
| Item 3 |  |  |  |  |  |  |
| BeL1&L1 | 15.38 | 14.04 | 6.81 | 7.05 | 41.42 | 75.43 |
| L2 | 19.22 | 14.49 | 8.56 | 6.12 | 36.52 | 20.74 |
| L3 | 21.43 | 25.36 | 8.75 | 6.79 | 38.77 | 28.44 |
| L4&L5 | 18.7 | 10.97 | 8.67 | 4.50 | 35.53 | 15.59 |
| Item 4 |  |  |  |  |  |  |
| BeL1&L1 | 18.08 | 16.58 | 3.53 | 5.45 | 28.45 | 27.80 |
| L2 | 21.56 | 17.99 | 5.25 | 10.19 | 29.77 | 24.88 |
| L3 | 21.61 | 16.28 | 5.15 | 6.56 | 28.51 | 19.04 |
| L4&L5 | 21.21 | 13.52 | 6.99 | 7.87 | 30.24 | 17.01 |
| Item 5 |  |  |  |  |  |  |
| BeL1&L1 | 35.06 | 52.35 | 1.49 | 8.23 | 54.05 | 58.17 |
| L2 | 48 | 39.39 | 3.12 | 8.04 | 71.10 | 52.35 |
| L3 | 55.3 | 35.76 | 4.56 | 6.76 | 78.79 | 53.45 |
| L4&L5 | 49.68 | 24.63 | 6.69 | 8.10 | 68.44 | 34.14 |
| Item 6 |  |  |  |  |  |  |
| BeL1&L1 | 21.57 | 23.47 | 3.34 | 7.41 | 31.27 | 27.82 |
| L2 | 24.8 | 18.03 | 3.00 | 3.52 | 32.35 | 21.36 |
| L3 | 25.54 | 24.07 | 3.86 | 3.38 | 32.35 | 25.45 |
| L4&L5 | 19.49 | 11.22 | 5.96 | 6.06 | 28.08 | 15.93 |
| Item 7 |  |  |  |  |  |  |
| BeL1&L1 | 25.66 | 40.77 | 1.49 | 2.09 | 40.47 | 48.94 |
| L2 | 37.65 | 34.71 | 3.64 | 7.33 | 59.28 | 43.30 |
| L3 | 35.57 | 28.00 | 5.14 | 7.53 | 65.56 | 35.76 |
| L4&L5 | 26.89 | 12.65 | 7.51 | 8.50 | 57.51 | 25.07 |

*Descriptive Statistics of Time Spent in Seconds on Each Item in Testlet L11 by Group*

**Table S5 (Continued)**

|  | Time to First Action | | Time for Last Action | | Total Time | |
| --- | --- | --- | --- | --- | --- | --- |
|  | Mean | SD | Mean | SD | Mean | SD |
| Item 8 |  |  |  |  |  |  |
| BeL1&L1 | 34.67 | 36.78 | 2.74 | 5.58 | 63.92 | 71.98 |
| L2 | 36.15 | 27.85 | 3.54 | 4.74 | 58.24 | 40.53 |
| L3 | 38.52 | 27.66 | 5.06 | 5.66 | 61.60 | 41.98 |
| L4&L5 | 39.77 | 32.36 | 6.81 | 8.86 | 62.57 | 44.26 |
| Item 9 |  |  |  |  |  |  |
| BeL1&L1 | 18.11 | 21.87 | 2.57 | 4.88 | 36.81 | 32.50 |
| L2 | 25.05 | 21.05 | 2.92 | 3.96 | 44.64 | 34.43 |
| L3 | 24.48 | 18.31 | 3.40 | 3.88 | 42.47 | 27.73 |
| L4&L5 | 22.35 | 12.28 | 3.75 | 3.64 | 40.48 | 33.47 |

*Descriptive Statistics of Time Spent in Seconds on Each Item in Testlet L11 by Group*

**Table S6**

|  | Time to First Action | | | Time for Last Action | | | Total Time | | |
| --- | --- | --- | --- | --- | --- | --- | --- | --- | --- |
|  | F | *p* | Pairwise t-value | F | *p* | Pairwise t-value | F | *p* | Pairwise t-value |
| Item 1 | 3.46 | **.016** |  | .45 | .715 |  | 2.59 | .052 |  |
| BeL1&L1 – L3 |  |  | 2.89* |  |  | — |  |  | — |
| Item 2 | 1.28 | .278 |  | 4.47 | **.004** |  | 1.37 | .249 |  |
| BeL1&L1 – L4&L5 |  |  | — |  |  | 3.28** |  |  | — |
| L3 – L4&L5 |  |  | — |  |  | 3.05* |  |  | — |
| Item 3 | .454 | .715 |  | 2.09 | .100 |  | .029 | .993 |  |
| Item 4 | 4.53 | **.004** |  | 15.66 | **<.001** |  | 8.91 | **<.001** |  |
| BeL1&L1 - L2 |  |  | 3.57** |  |  | 3.00* |  |  | 4.97*** |
| BeL1&L1 – L3 |  |  | 2.98* |  |  | 5.21*** |  |  | 4.61*** |
| BeL1&L1 – L4&L5 |  |  | 3.09* |  |  | 6.11*** |  |  | 4.16*** |
| L2 – L3 |  |  | — |  |  | 3.11* |  |  | — |
| L2 – L4&L5 |  |  | — |  |  | 4.43*** |  |  | — |
| Item 5 | 5.91 | **<0.001** |  | 7.85 | **<0.001** |  | 10.63 | **<.001** |  |
| BeL1&L1 - L2 |  |  | — |  |  | — |  |  | 4.37*** |
| BeL1&L1 – L3 |  |  | — |  |  | 3.93** |  |  | 5.65*** |
| BeL1&L1 – L4&L5 |  |  | — |  |  | 4.25*** |  |  | 3.94*** |
| L2 – L4&L5 |  |  | -4.10*** |  |  | 2.84* |  |  | — |
| Item 6 | 6.59 | **<.001** |  | 9.40 | **<0.001** |  | 3.54 | **.014** |  |
| BeL1&L1 – 3 |  |  | — |  |  | 3.36** |  |  | 2.88* |
| BeL1&L1 – L4&L5 |  |  | 4.41*** |  |  | 4.64*** |  |  | — |
| 2 – L4&L5 |  |  | 3.04* |  |  | 4.09*** |  |  | — |
| 3 – L4&L5 |  |  | 2.97* |  |  | — |  |  | — |

*One-Way ANOVA of Time Spent in Seconds on Each Item in Testlet L12 by Group*

*Note:* Pairwise comparisons estimated using the Bonferroni correction; ****p*<.001; **<.001; *<.05

**Table S6 (Continued)**

|  | Time to First Action | | | Time for Last Action | | | Total Time | | |
| --- | --- | --- | --- | --- | --- | --- | --- | --- | --- |
|  | F | *p* | Pairwise t-value | F | *p* | Pairwise t-value | F | *p* | Pairwise t-value |
| Item 7 | 4.33 | **<.001** |  | 9.04 | **<.001** |  | 3.49 | **.015** |  |
| BeL1&L1 - L2 |  |  | 3.32** |  |  | — |  |  | 3.15* |
| BeL1&L1 – L3 |  |  | 2.74* |  |  | 3.64** |  |  | 2.82* |
| BeL1&L1 – L4&L5 |  |  | 3.26** |  |  | 4.14*** |  |  | — |
| L2 – L3 |  |  | — |  |  | 3.15* |  |  | — |
| L2 – L4&L5 |  |  | — |  |  | 3.69** |  |  | — |
| Item 8 | 14.72 | **<.001** |  | 12.30 | **<.001** |  | 7.39 | **<.001** |  |
| BeL1&L1 – L3 |  |  | 5.45*** |  |  | 4.79*** |  |  | 4.63*** |
| BeL1&L1 – L4&L5 |  |  | 6.48*** |  |  | 5.87*** |  |  | 4.03*** |
| BeL1&L1 – L4&L5 |  |  | 5.50*** |  |  | — |  |  | 3.02* |
| L2 – L3 |  |  | — |  |  | 5.42*** |  |  | — |
| L2 – L4&L5 |  |  | — |  |  | 6.38*** |  |  | — |
| Item 9 | 24.26 | **<.001** |  | 5.20 | **.001** |  | 25.02 | **<.001** |  |
| BeL1&L1 - L2 |  |  | 3.52** |  |  | — |  |  | 4.10*** |
| BeL1&L1 – L3 |  |  | 6.66*** |  |  | 3.22** |  |  | 6.45*** |
| BeL1&L1 – L4&L5 |  |  | 7.27*** |  |  | 3.67** |  |  | 8.02*** |
| L2 – L3 |  |  | 4.44*** |  |  | — |  |  | 3.28** |
| L2 – L4&L5 |  |  | 5.33*** |  |  | — |  |  | 5.63*** |
| L3 – L4&L5 |  |  | — |  |  | — |  |  | 3.19** |

*One-Way ANOVA of Time Spent in Seconds on Each Item in Testlet L12 by Group*

*Note:* Pairwise comparisons estimated using the Bonferroni correction; ****p*<.001; **<.001; *<.05

**Table S7**

|  | Time to First Action | | | Time for Last Action | | | Total Time | | |
| --- | --- | --- | --- | --- | --- | --- | --- | --- | --- |
|  | F | *p* | Pairwise t-value | F | *p* | Pairwise t-value | F | *p* | Pairwise t-value |
| Item 1 | 0.99 | .397 |  | .61 | .610 |  | 1.23 | .296 |  |
| Item 2 | 6.32 | **<.001** |  | 16.42 | **<.001** |  | 3.93 | **.008** |  |
| BeL1&L1 – L3 |  |  | 3.32** |  |  | 2.88* |  |  | — |
| BeL1&L1 –L4&L5 |  |  | 4.31*** |  |  | 5.29*** |  |  | — |
| L2 – L3 |  |  | 3.47** |  |  | 2.87* |  |  | — |
| L2 – L4&L5 |  |  | — |  |  | 6.34*** |  |  | — |
| L3 – L4&L5 |  |  | — |  |  | 4.29*** |  |  | — |
| Item 3 | 18.49 | **<.001** |  | 8.24 | **<.001** |  | 17.21 | **<.001** |  |
| BeL1&L1 - L2 |  |  | 4.00*** |  |  | — |  |  | 3.02** |
| BeL1&L1 – L3 |  |  | 5.96*** |  |  | 2.75* |  |  | 5.44*** |
| BeL1&L1 –L4&L5 |  |  | 6.67*** |  |  | 4.23*** |  |  | 5.87*** |
| L2 – L3 |  |  | 3.27** |  |  | — |  |  | 4.12*** |
| L2 – L4&L5 |  |  | 4.46*** |  |  | 4.05*** |  |  | 4.63*** |
| L3 – L4&L5 |  |  | — |  |  | 2.76* |  |  | — |
| Item 4 | 20.25 | **<.001** |  | 12.98 | **<.001** |  | 9.44 | **<.001** |  |
| BeL1&L1 – 3 |  |  | 5.32*** |  |  | — |  |  | 3.35** |
| BeL1&L1 –L4&L5 |  |  | 5.69*** |  |  | 4.40*** |  |  | 3.73** |
| L2 – L3 |  |  | 5.28*** |  |  | 3.25** |  |  | 3.70** |
| L2 – L4&L5 |  |  | 5.41*** |  |  | 5.68*** |  |  | 3.94*** |
| L3 – L4&L5 |  |  |  |  |  | 3.27** |  |  | — |
| Item 5 | 4.86 | **.002** |  | — | — |  | .20 | .896 |  |
| BeL1&L1 – L3 |  |  | -2.65* |  |  | — |  |  | — |
| BeL1&L1 –L4&L5 |  |  | -3.16** |  |  | — |  |  | — |
| L2 – L4&L5 |  |  | -2.74* |  |  | — |  |  | — |

*One-Way ANOVA of Time Spent in Seconds on Each Item in Testlet L13 by Group*

*Note:* Pairwise comparisons estimated using the Bonferroni correction; ****p*<.001; **<.001; *<.05.

**Table S7 (Continued)**

|  | Time to First Action | | | Time for Last Action | | | Total Time | | |
| --- | --- | --- | --- | --- | --- | --- | --- | --- | --- |
|  | F | *p* | Pairwise t-value | F | *p* | Pairwise t-value | F | *p* | Pairwise t-value |
| Item 6 | 3.81 | **.009** |  | 8.61 | **<.001** |  | 3.77 | **.010** |  |
| BeL1&L1 – L3 |  |  | 3.18** |  |  | — |  |  | 3.02** |
| BeL1&L1 – L4 |  |  | — |  |  | 4.14*** |  |  | — |
| L2 – L4&L5 |  |  | — |  |  | 4.37*** |  |  | — |
| L3 – L4&L5 |  |  | — |  |  | 3.18** |  |  | — |
| Item 7 | 4.06 | **.007** |  | 8.32 | **<.001** |  | 4.78 | **.002** |  |
| BeL1&L1 - L2 |  |  | 2.90* |  |  | — |  |  | — |
| BeL1&L1 – L3 |  |  | 3.38** |  |  | — |  |  | 3.10** |
| BeL1&L1 – L4&L5 |  |  | — |  |  | 3.32** |  |  | — |
| L2 – L4&L5 |  |  | — |  |  | 4.47*** |  |  | — |
| L3 – L4&L5 |  |  | — |  |  | 4.36*** |  |  | — |
| Item 8 | 6.19 | **<.001** |  | .77 | .514 |  | 5.25 | **.001** |  |
| BeL1&L1 - L2 | 3.31 |  | 3.31** |  |  | — |  |  | — |
| BeL1&L1 – L3 |  |  | 3.43** |  |  | — |  |  | 3.38** |
| L3 – L4&L5 |  |  | -2.73* |  |  | — |  |  | — |
| Item 9 | 2.38 | .068 |  | 6.29 | **<.001** |  | 7.56 | **<.001** |  |
| BeL1&L1 - L2 |  |  | — |  |  | — |  |  | — |
| BeL1&L1 – L3 |  |  | — |  |  | — |  |  | 3.55** |
| BeL1&L1 – L4 |  |  | — |  |  | 3.66** |  |  | 4.27*** |
| L2 – L4&L5 |  |  | — |  |  | 3.57** |  |  | 3.15* |
| L3 – L4&L5 |  |  | — |  |  | 3.21** |  |  | — |

*One-Way ANOVA of Time Spent in Seconds on Each Item in Testlet L13 by Group*

*Note:* Pairwise comparisons estimated using the Bonferroni correction; ****p*<.001; **<.001; *<.05

**Table S8**

|  | Time to First Action | | | Time to Last Action | | | Total Time | | |
| --- | --- | --- | --- | --- | --- | --- | --- | --- | --- |
|  | F | *p* | Pairwise T-value | F | *p* | Pairwise T-value | F | *p* | Pairwise T-value |
| Testlet L11 | 5.22 | .001 |  | 24.34 | <.001 |  | 1.22 | .300 |  |
| BeL1&L1 – L2 |  |  | 2.80* |  |  | 3.28** |  |  | 0.574 |
| BeL1&L1 – L3 |  |  | 3.82*** |  |  | 5.22*** |  |  | 0.375 |
| BeL1&L1 – L4&L5 |  |  | 1.80 |  |  | 8.03*** |  |  | 0.986 |
| L2 – L3 |  |  | 1.30 |  |  | 2.57 |  |  | 0.979 |
| L2 – L4&L5 |  |  | -0.84 |  |  | 6.43*** |  |  | 0.753 |
| L3 – L4&L5 |  |  | -1.91 |  |  | 4.69*** |  |  | 0.531 |
| Testlet L12 | 5.34 | <.001 |  | 29.42 | <.001 |  | 13.03 | <.001 |  |
| BeL1&L1 – L2 |  |  | 5.34*** |  |  | 3.53** |  |  | 5.50*** |
| BeL1&L1 – L3 |  |  | 5.99*** |  |  | 6.45*** |  |  | 5.88*** |
| BeL1&L1 – L4&L5 |  |  | 5.20*** |  |  | 8.39*** |  |  | 5.41*** |
| L2 – L3 |  |  | .78 |  |  | 4.12*** |  |  | .36 |
| L2 – L4&L5 |  |  | .55 |  |  | 6.77*** |  |  | .64 |
| L3 – L4&L5 |  |  | -.04 |  |  | 3.69** |  |  | .37 |
| Testlet L13 | 12.17 | <.001 |  | 25.69 | <.001 |  | 8.75 | <.001 |  |
| BeL1&L1 – L2 |  |  | 4.19*** |  |  | 1.86 |  |  | 3.21** |
| BeL1&L1 – L3 |  |  | 5.82*** |  |  | 3.75** |  |  | 4.84*** |
| BeL1&L1 – L4&L5 |  |  | 4.88*** |  |  | 6.85*** |  |  | 3.74** |
| L2 – L3 |  |  | 2.69* |  |  | 3.23** |  |  | 2.73* |
| L2 – L4&L5 |  |  | 1.43 |  |  | 7.80*** |  |  | 1.09 |
| L3 – L4&L5 |  |  | -.76 |  |  | 5.53*** |  |  | -1.15 |

*One-Way ANOVA of Timing Variables Averaged Across Items for Each Testlet by Group*

*Note:* Pairwise comparisons estimated using the Bonferroni correction; ****p*<.001; **<.001; *<.05

**Table S9**

*Descriptive Statistics of Timing Variables Across Literacy Proficiency Group for Testlets L12 and L13*

|  | Time to First Action | | | | Time for Last Action | | | | Total Time | | | |
| --- | --- | --- | --- | --- | --- | --- | --- | --- | --- | --- | --- | --- |
|  | Mean | *SD* | Bottom 5% | Top 5% | Mean | *SD* | Bottom 5% | Top 5% | Mean | *SD* | Bottom 5% | Top 5% |
| Testlet L12 |  |  |  |  |  |  |  |  |  |  |  |  |
| Total | 38.38 | 19.66 | 13.63 | 73.90 | 4.31 | 3.52 | 1.11 | 11.16 | 61.16 | 29.49 | 24.47 | 116.77 |
| Below Level 1  & Level 1 | 26.93 | 16.28 | 4.35 | 58.27 | 2.25 | 1.70 | .20 | 5.69 | 43.83 | 23.24 | 10.40 | 81.06 |
| Level 2 | 39.05 | 20.30 | 13.76 | 73.87 | 3.65 | 3.37 | 1.09 | 10.63 | 62.55 | 31.91 | 25.22 | 123.31 |
| Level 3 | 40.22 | 20.15 | 16.17 | 75.03 | 4.74 | 3.38 | 1.36 | 11.29 | 63.37 | 29.18 | 27.70 | 117.71 |
| Levels 4 & 5 | 40.14 | 16.60 | 21.47 | 74.25 | 5.97 | 4.07 | 1.74 | 13.24 | 64.42 | 24.98 | 34.11 | 116.19 |
| Testlet L13 |  |  |  |  |  |  |  |  |  |  |  |  |
| Total | 45.71 | 21.91 | 14.17 | 84.26 | 4.37 | 4.25 | .89 | 13.10 | 72.44 | 35.46 | 26.48 | 131.65 |
| Below Level 1  & Level 1 | 31.75 | 19.99 | 1.72 | 68.09 | 2.41 | 1.94 | .23 | 5.92 | 54.25 | 30.14 | 6.87 | 107.88 |
| Level 2 | 44.09 | 25.01 | 11.22 | 90.87 | 3.45 | 3.65 | .75 | 10.61 | 69.65 | 37.22 | 22.02 | 135.96 |
| Level 3 | 48.45 | 20.78 | 22.41 | 84.51 | 4.45 | 4.05 | 1.16 | 13.16 | 76.86 | 37.45 | 33.98 | 135.33 |
| Levels 4 & 5 | 46.99 | 17.06 | 27.61 | 77.80 | 6.47 | 5.29 | 1.72 | 17.82 | 73.26 | 25.68 | 40.73 | 126.01 |

*Note:* *SD* = Standard Deviation; Bottom and Top 5% = average timing of respondents in the 5^th^ and 95^th^ percentile of the distribution.

**Table S10**

|  | Time to First Action | | Time for Last Action | | Total Time | |
| --- | --- | --- | --- | --- | --- | --- |
|  | Mean | *SD* | Mean | *SD* | Mean | *SD* |
| Item 1 |  |  |  |  |  |  |
| Cluster 1 | 44.36 | 16.35 | 11.40 | 11.43 | 66.35 | 25.41 |
| Cluster 2 | 19.36 | 79.32 | 6 | 17.98 | 33.51 | 84.89 |
| Item 2 |  |  |  |  |  |  |
| Cluster 1 | 39.40 | 12.38 | 6.78 | 5.18 | 66.63 | 24.56 |
| Cluster 2 | 18.52 | 34.80 | 3.07 | 9.13 | 35.81 | 49.54 |
| Item 3 |  |  |  |  |  |  |
| Cluster 1 | 26.33 | 10.94 | 11.69 | 4.39 | 57.89 | 16.80 |
| Cluster 2 | 15.22 | 19.17 | 6.78 | 9.20 | 30.53 | 74.61 |
| Item 4 |  |  |  |  |  |  |
| Cluster 1 | 33.44 | 10.54 | 8.44 | 5.03 | 47.80 | 17.77 |
| Cluster 2 | 15.76 | 24.77 | 3.47 | 15.20 | 22.53 | 33.48 |
| Item 5 |  |  |  |  |  |  |
| Cluster 1 | 87.92 | 24.57 | 5.17 | 5.58 | 127.70 | 29.54 |
| Cluster 2 | 28.64 | 53.75 | 1.78 | 12.42 | 44.10 | 59.93 |
| Item 6 |  |  |  |  |  |  |
| Cluster 1 | 38.19 | 13.17 | 4.17 | 4.93 | 48.86 | 16.46 |
| Cluster 2 | 18.75 | 26.46 | 2.70 | 4.54 | 25.94 | 30.03 |
| Item 7 |  |  |  |  |  |  |
| Cluster 1 | 66.18 | 18.74 | 4.37 | 6.77 | 96.34 | 27.29 |
| Cluster 2 | 22.82 | 52.36 | 2.59 | 5.25 | 38.90 | 57.88 |
| Item 8 |  |  |  |  |  |  |
| Cluster 1 | 60.60 | 22.07 | 6.13 | 2.57 | 100.57 | 32.65 |
| Cluster 2 | 26.61 | 36.06 | 2.31 | 8.06 | 44.84 | 67.18 |
| Item 9 |  |  |  |  |  |  |
| Cluster 1 | 37.28 | 16.25 | 4.83 | 2.22 | 70.97 | 23.82 |
| Cluster 2 | 17.80 | 26.15 | 2.05 | 6.85 | 31.82 | 39.84 |

*Mean Time in Seconds Spent in Testlet L11 on Each Item by Cluster*

*Note:* t-tests reveal significant differences between the clusters on all items across all timing variables (*ps* < .001).

**Table S11**

*Demographic Frequencies for Respondents with Missing and Non-Missing Skills-Use at Home Data*

|  | Missing Skills-Use | | Non-Missing Skills-Use | |
| --- | --- | --- | --- | --- |
| Variable | Frequency | % | Frequency | % |
| Total | 87 |  | 272 |  |
| Learning Disability | 13 | 15% | 23 | 8% |
| Education |  |  |  |  |
| Less than High School | 18 | 21% | 41 | 15% |
| High School | 47 | 54% | 138 | 51% |
| More High School | 22 | 25% | 93 | 34% |
| Age |  |  |  |  |
| Younger | 37 | 43% | 131 | 48% |
| Middle | 14 | 16% | 48 | 18% |
| Older | 36 | 41% | 93 | 34% |
| Employment |  |  |  |  |
| Employed | 69 | 79% | 185 | 68% |
| Unemployed | 3 | 3% | 38 | 14% |
| Out of Labor Force | 15 | 17% | 49 | 18% |
| Native Speaker | 69 | 79% | 214 | 79% |
| Race |  |  |  |  |
| White | 47 | 54% | 147 | 54% |
| Hispanic | 18 | 21% | 54 | 20% |
| Black | 14 | 16% | 50 | 18% |
| Asian | 5 | 6% | 15 | 6% |

*Note*: Percentages indicate the proportion of individuals in each demographic category relative to the total number of individuals in each cluster.

**Table S12**

|  | logit | Odds Ratio | 95% CI | *p* |
| --- | --- | --- | --- | --- |
| (Intercept) | -1.04 | .35 | .12 – 1.03 | .058 |
| Literacy Score | -.45 | .64 | .50 – .81 | **<.001** |
| Learning Disability | .59 | 1.8 | .87 – 3.64 | .107 |
| Education ^a^ |  |  |  |  |
| High school | -.24 | .79 | .42 – 1.50 | .456 |
| More than high school | -.61 | .54 | .26 – 1.14 | .104 |
| Age ^b^ |  |  |  |  |
| Middle age | -.02 | .98 | .49 – 1.92 | .962 |
| Older age | .35 | 1.41 | .82 – 2.45 | .216 |
| Employment ^c^ |  |  |  |  |
| Not Employed | -1.77 | .17 | .05 – 0.44 | **.001** |
| Out of the labor force | -.47 | .63 | .33 – 1.13 | .131 |
| Native Speaker | .27 | 1.3 | .58 – 2.98 | .523 |
| Race and Ethnicity ^d^ |  |  |  |  |
| Hispanic | .13 | 1.14 | .50 – 2.54 | .746 |
| Black | -.26 | .77 | .41 – 1.42 | .419 |
| Asian | .26 | 1.3 | .39 – 4.04 | .657 |

*Regression Results of Literacy Scores and Demographic Factors Predicting Missingness on Skills-Use at Home*

*Note:* The findings are derived from respondents who scored a Level 2 or below in Testlet L11. Cases without missing data are the reference category. 95% CI = 95% confidence interval. ^a^ Less than high school is the comparison. ^b^ Younger age is the comparison. ^c^ Employed is the comparison. ^d^ White is the comparison.

**Table S13**

*Descriptive Statistics of Skills-Use and Literacy Scores across Cluster Membership for Respondents with Complete Data*

|  | Cluster 1 | | | | Cluster 2 | | | |
| --- | --- | --- | --- | --- | --- | --- | --- | --- |
| Variable | *N* | Mean | *SD* | Min – Max | *N* | Mean | *SD* | Min – Max |
| Skills-Use |  |  |  |  |  |  |  |  |
| ICT at Home | 74 | 1.96 | .90 | .05 – 1.62 | 198 | 2.10 | 1.05 | -.27 – 4.22 |
| Reading at Home | 74 | 2.74 | .66 | 1.38 – 4.69 | 198 | 2.59 | .98 | .31 – 7.43 |
| Writing at Home | 74 | 2.50 | .96 | -.30 – 5.03 | 198 | 2.22 | 1.05 | -.30 – 4.95 |
| Numeracy at Home | 74 | 2.40 | .92 | .17 – 5.28 | 198 | 2.35 | .93 | .14 – 6.17 |
| Literacy Score | 74 | 251.41 | 22.01 | 184.65 – 299.54 | 198 | 242.16 | 27.02 | 150.40 – 298.12 |

*Note:* *N* = Number of respondents in the entire cluster with complete data. *SD* = Standard Deviation; Min-Max = Minimum – Maximum.

**Table S14**

| Variable | Cluster 1 | | Cluster 2 | |
| --- | --- | --- | --- | --- |
|  | Frequency | % | Frequency | % |
| Total | 74 |  | 198 |  |
| Learning Disability | 6 | 8% | 17 | 9% |
| Education |  |  |  |  |
| Less than High School | 11 | 15% | 30 | 15% |
| High School | 32 | 43% | 106 | 54% |
| More High School | 31 | 42% | 62 | 31% |
| Age |  |  |  |  |
| Younger | 19 | 26% | 43 | 22% |
| Middle | 11 | 15% | 37 | 19% |
| Older | 44 | 59% | 49 | 25% |
| Employment |  |  |  |  |
| Employed | 46 | 62% | 139 | 70% |
| Unemployed | 13 | 18% | 25 | 13% |
| Out of Labor Force | 15 | 20% | 34 | 17% |
| Native Speaker | 56 | 76% | 158 | 80% |
| Race |  |  |  |  |
| White | 38 | 51% | 105 | 53% |
| Hispanic | 11 | 15% | 43 | 27% |
| Black | 19 | 26% | 31 | 16% |
| Asian | 6 | 8% | 9 | 5% |

*Demographic Frequencies across Cluster Membership for Respondents with Complete Data*

*Note*: Percentages indicate the proportion of individuals in each demographic category relative to the total number of individuals in each cluster.

**Table S15**

|  | logit | Odds Ratio | *95% CI* | *p* |
| --- | --- | --- | --- | --- |
| (Intercept) | 2.78 | 16.12 | 3.44 – 81.98 | **<.001** |
| Skills-Use |  |  |  |  |
| ICT Home | .37 | 1.45 | .94 – 2.27 | .099 |
| Reading at Home | .13 | 1.14 | .72 – 1.84 | .582 |
| Writing at Home | -.55 | .58 | .36 – 0.91 | **.020** |
| Numeracy at Home | -.19 | .83 | .55 – 1.24 | .364 |
| Literacy Score | -.80 | .45 | .26 – 0.92 | **.036** |
| Learning Disability | -1.24 | .29 | .08 – 1.13 | .065 |
| Education ^a^ |  |  |  |  |
| High school | .44 | 1.55 | .58 – 4.05 | .376 |
| More than high school | .31 | 1.36 | .46 – 4.00 | .576 |
| Age ^b^ |  |  |  |  |
| Middle age | -1.07 | .34 | .13 – 0.93 | **.033** |
| Older age | -2.56 | .08 | .03 – 0.19 | **<.001** |
| Employment ^c^ |  |  |  |  |
| Not Employed | -.94 | .39 | .16 – 0.97 | **.042** |
| Out of the labor force | -.72 | .49 | .21 – 1.15 | .099 |
| Native Speaker | .35 | 1.42 | .48 – 4.39 | .527 |
| Race and Ethnicity ^d^ |  |  |  |  |
| Hispanic | -.66 | .51 | .15 – 1.84 | .292 |
| Black | -1.62 | .20 | .08 – 0.49 | **<.001** |
| Asian | -1.33 | .26 | .05 – 1.36 | .104 |

*Individual and Contextual Factors Predicting Cluster Membership for Respondents with Complete Data*

*Note: N* = 272*;* Cluster 1 is the reference category. 95% CI = 95% Confidence Interval. ^a^ Less than high school is the comparison. ^b^ Younger age is the comparison. ^c^ Employed is the comparison. ^d^ White is the comparison.

**Figure S1**

*
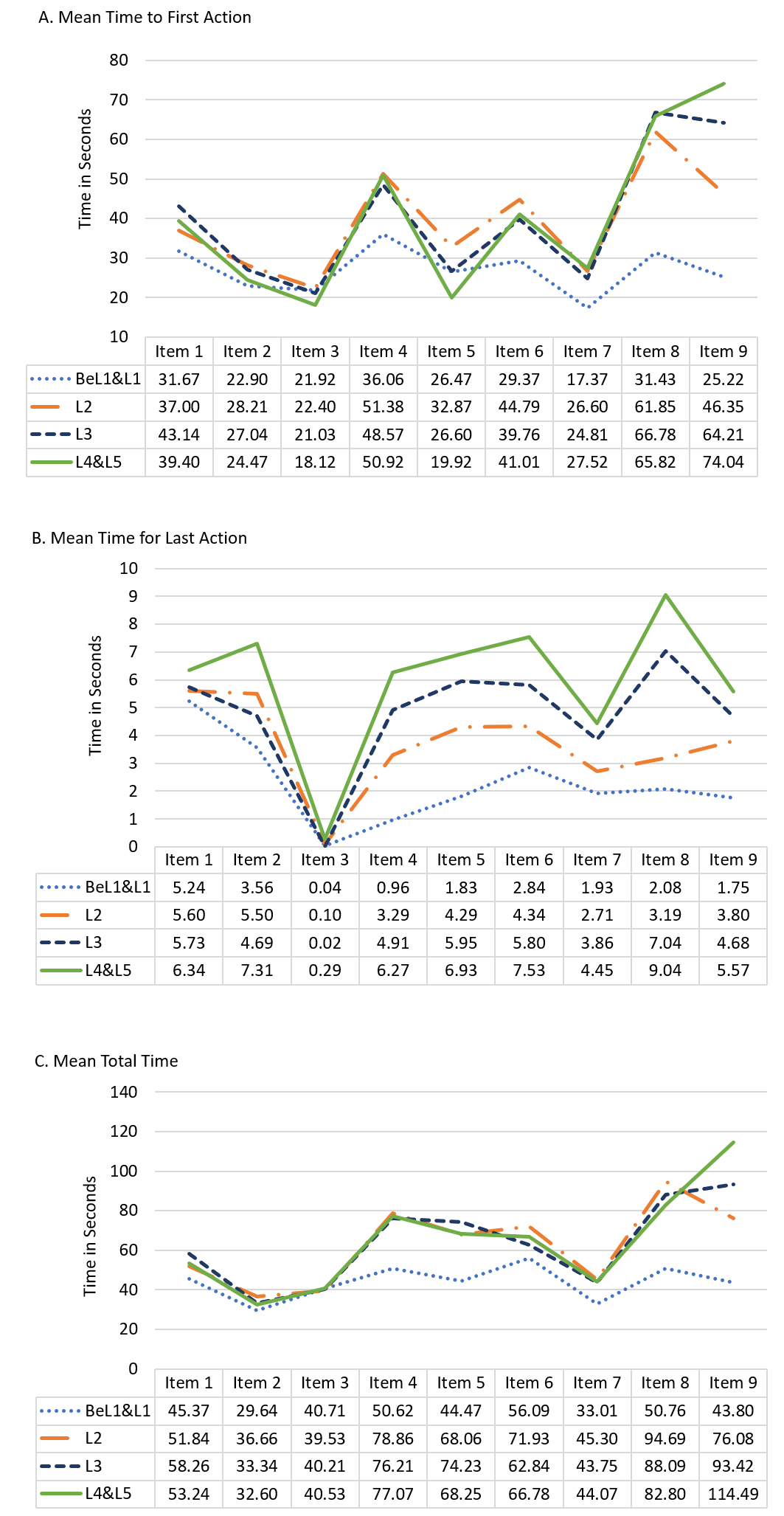
Timing Variables by Literacy Proficiency Group in Testlet L12*

**Figure S2**

*
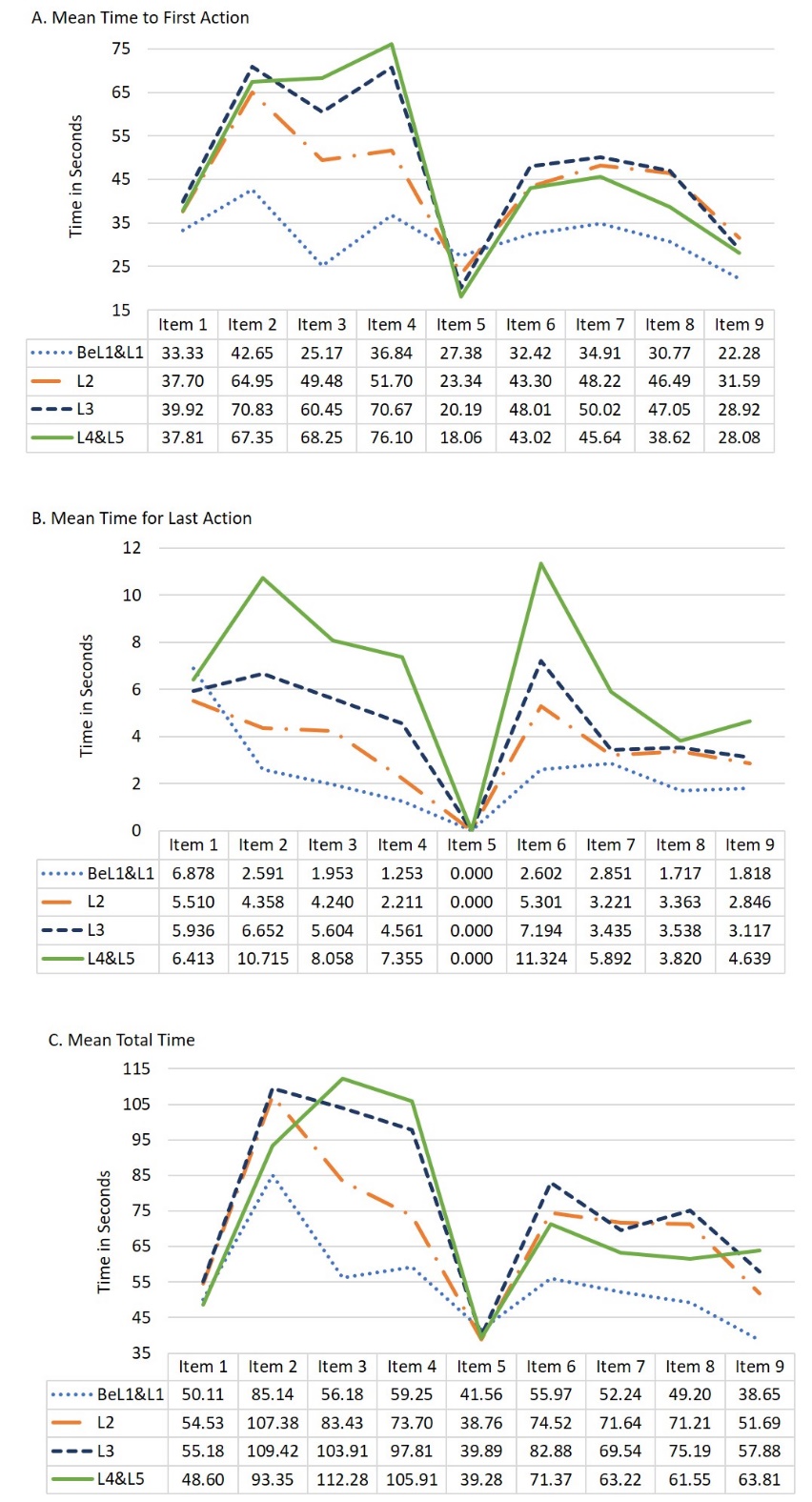
Timing Variables by Literacy Proficiency Group in Testlet L13*

**Figure S3***Silhouette Scores for Clusters Solutions with 2 through 8 Clusters*

**
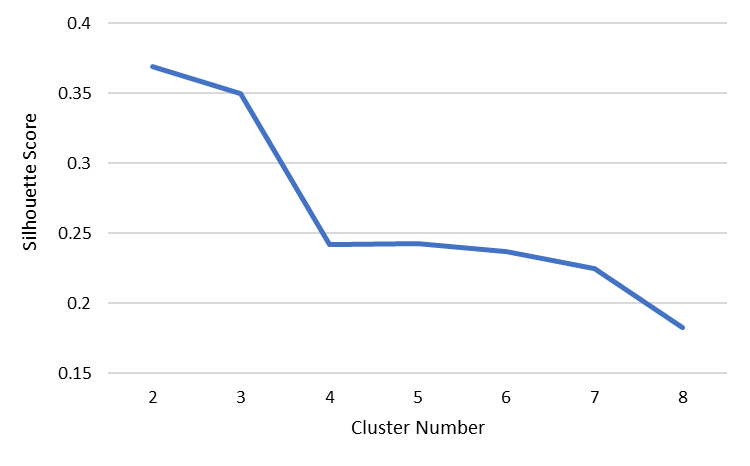
**
